# Exploring the potential of nano-enhanced lemongrass biodiesel blends with hydrogen premixing for improved CI engine behaviour

**DOI:** 10.1038/s41598-025-16786-9

**Published:** 2025-10-30

**Authors:** Krupakaran Radhakrishnan Lawrence, Praveen Anchupogu, Ratna Kamala Petla, Dhinesh Balasubramanian, Utku Kale, Artūras Kilikevičius

**Affiliations:** 1 Department of Mechanical Engineering, Mohan Babu University, Tirupati, Andhra Pradesh India; 2 Department of Mechanical Engineering, Bapatla Engineering College, Bapatla, Andhra Pradesh India; 3 Department of Electronics and Communication Engineering, Siddharth Institute of Science & Technology Puttur, Puttur, Andhra Pradesh India; 4 Department of Mechanical Engineering, Mepco Schlenk Engineering College, Sivakasi, Tamil Nadu India; 5 Department of Port Engineering, Lithuanian Maritime Academy (LMA), Vilnius Gediminas Technical University, Klaipėda, Lithuania; 6 Department of Aeronautics and Naval Architecture, Faculty of Transportation Engineering and Vehicle Engineering, Budapest University of Technology and Economics, Műegyetem rkp. 3, Budapest, H-1111 Hungary; 7 Mechanical Science Institute, Vilnius Gediminas Technical University, Plytinės g. 25, Vilnius, LT-10105 Lithuania

**Keywords:** Hydrogen, Lemongrass oil, MWCNTs, Engine performance characteristics, Energy science and technology, Engineering

## Abstract

Using fossil fuels in compression ignition (CI) engines is still a challenge today due to concerns over environmental pollution and the long-term sustainability of fuel sources. Addressing these difficulties necessitates the development of alternate fuel methods that can boost engine performance while simultaneously lowering emissions. The current study focusses on employing a 20% blend of lemongrass (Cymbopogon Citratus) biodiesel and diesel supplemented with MWCNTs at 40 ppm (also known as “nano fuel of Lemongrass oil”) and investigating the usage of this mixture in an unmodified CI engine. To improve the performance of the nano-fuel, 5LPM hydrogen was premixed through intake manifold. This led to significantly better combustion, thanks to improved mixing inside the cylinder and quicker flame spread. Notably, adding 5 LPM hydrogen to the nano additive biodiesel made from lemongrass biodiesel resulted in a 12.6% boost in brake thermal efficiency. When compared to the LG20 blend operation, these studies revealed a 15.34% reduction in brake-specific fuel consumption (BSFC), a 19.6% decrease in carbon monoxide (CO) pollutants, a 5.35% decrease in unburned hydrocarbon (UHC) pollutants, and an 8.33% reduction in smoke opacity. As a result, we propose further research into hydrogen premixing in conjunction with nano-enhanced Lemongrass biodiesel to increase CI engine performance. It has been noticed that LG20 + MC + H2 fuel increases BTE by 12.6% and reduces BSFC by 15.33% when compared to LG20 fuel. These findings demonstrate the synergistic effect of hydrogen premixing and nanoparticle-enriched biodiesel in increasing CI engine performance and lowering emissions, indicating a possible path towards cleaner and more efficient engine operation.

## Introduction

The world’s energy demand is currently increasing rapidly, and a large percentage of that consumption is based on fossil fuels, which are depleting and polluting the environment. CI (compression ignition) engines are the most efficient locomotives utilised in power generation, agriculture, and automotive industries because of their remarkable performance, longevity, and reliability. The harmful exhaust gases such as NO_x_, CO, CO_2,_ and particulate matter generated by the diesel engines affect the environment and human health, and cause global warming, so that recently many countries have imposed stringent laws and norms for reducing environmental pollution. Because of increasing concerns about the environmental and economic consequences of petroleum-based fuels, researchers have aggressively investigated and found a variety of alternative sources to replace traditional fossil fuels. The key problems in diesel engines for reducing emissions and improving combustion were fuel modification, engine design changes, and the employment of exhaust gas after-treatment technologies. Many researchers have worked on these methods, but hydrogen was considered the most appropriate substitute fuel due to its easy production and its ability to curtail the exhaust of a diesel engine. Biofuels produced by various sources were becoming a substantial fuel to attain the present energy demand in the automobile sector due to their merits, such as biodegradable, renewable, and environmentally friendly fuels in comparison with petroleum fuels.

Rajak et al. investigated the effect of fuel blends (diethyl ester fuels, hydrogen-added diesel, Spirulina microalgae and n-butanol) and analysed engine performance characteristics^1^. Adding hydrogen to fuels, enhances the BTE (0.95%), NO_x_ (21.3%) at peak loads. In addition, CO_2_, BSN (22.89%) emissions, and SFC (18.3%) were minimized with the addition of hydrogen. Gad et al. synthesised HHO gas through electrolysis and blended it with cotton ethyl ester blends to examine the output of diesel engine fuelled with kerosene as an addition for cold starting. Following experimenting, 17% increased BTE and 26.2% decreased BSFC for HHO petrol, kerosene and biodiesel blends compared to the B20 fuel. Decrements in EGT, CO_2_, and CO by 20.6%, 11.7%, and 11% were also observed, with an increment of 14% for NO_x_ emissions by adding HHO gas and 5% kerosene. Finally, enriching HHO enhances combustion, and kerosene enhances the cold flow characteristics of biodiesel^2^. Koten was discovered an improvement in BTE and BSFC with the addition of hydrogen through the intake port of the CI engine with varied flow ratios (0.20, 0.40, 0.60, and 0.80 lpm). The outlet gas temperature and NO_x_ emissions increase (at 0.80 lpm dramatic increment) at peak loads, but SOOT, UHC and CO pollutants declined considerably for hydrogen gas as supplementary fuel at every load^3^. Yaliwal et al. have investigated the renewable fuels, which include Honge methyl ester (HOME) and producer gas, providing advantages including biodegradability and energy security, rendering them appropriate for both energy production and transportation. A study adjusted the nozzle design and combustion chamber arrangements in a dual-fuel diesel engine, resulting in higher performance with lower emissions. The best results were obtained using a re-entrant combustion chamber, 230 bar injection pressure, and a 4-hole, 0.25 mm nozzle, resulting in a 4–5% boost in efficiency. Additional research is needed to improve dual-fuel operation^4^. Singh et al. studied the Utilization of fossil fuels causes environmental problems and diminishes resources, emphasizing the importance of alternative energy. This study looks at the usage of aamla seed oil biodiesel (AB) and eucalyptus oil (EU) mixes in compression ignition (CI) engines. The findings indicate that a 70% Aamla biodiesel 30% Eucalyptus oil mix (AB70EU30) can successfully substitute diesel, provide comparable performance while lower CO, HC, and smoke pollutants, with NOx values comparable to diesel^5^. Jegadheesan et al. conducted engine tests with Pongamia pinnata biodiesel with hydrogen as an inducted fuel in a CI engine. At 80% load condition, 0.33 and 3.24% increments of BTE were noted with 10 lpm hydrogen and biodiesel compared to diesel and biodiesel. Further, HC emissions decreased by 13 ppm, CO and CO_2_ emissions reduced by 0.02 and 3.8% compared to biodiesel^6^. Khayum et al. utilised dual fuel mode diesel engine (raw biogas-WCOME) to analyse an engine with various nozzle opening pressures. Thermal efficiency increased by 4.6% with 240 bar injection pressure. When operating with dual fuels, NO emissions grew as the nozzle opening pressure increased, but they remained lower than when using diesel fuel^7^. Ambarita et al.^8^ explored the influence of the flow rate of biogas and the concentration level of methane on the engine behavior powered with dual-fuel mode. The output power and SFC were superior to those in diesel mode, powered with dual fuel mode. Maximum BTE was obtained with a load of 1500 W and rated speed of 1500 rpm with optimum biogas flow rate (15% and 18%) and methane concentrations (60 and 70%)^8^. Zhang et al. conducted experiments on high high-pressure, six-cylinder common rail diesel engine to assess the BTE and NOx emissions with diesel-LNG dual fuel mode. They suggested that optimum BTE and lowered NOx emissions were obtained with altering the diesel injection timing in retard (i.e. 1–2 CA ATDC) condition^9^. Britto and Martins were analyzed the engines, primarily driven by diesel, were critical for operations worldwide, however, alternatives to gasoline in dual-fuel systems provide issues due to inadequate data. This experiment utilised a diesel engine with ethanol in dual-fuel operation mode to optimize calibration for fuel economy. Several parameters, which include compression ratios, injector flow rates, and diesel injection pressures, were examined to determine performance and the diesel impact of substitution^10^. Castro et al. studied the engine parameters of turbo turbocharged 4 4-cylinder DI engine powered with diesel with hydrogen. With the replacement of hydrogen (80%), diesel fuel consumption was reduced by 54.2–30% of engine load. CO_2_ and smoke opacity were minimized in all conditions, but at lower engine loads, NO_x_ pollutants were also reduced with hydrogen addition^11^. Aldhaidhawi et al. performed both experimental and numerical studies on the influence of rapeseed biodiesel (B20) with hydrogen content on the formation of the mixture and engine parameters of the tractor CI engine. They discovered that injecting hydrogen into the intake air flow with B20 fuel reduces smoke, total UBHC, and CO emissions while maintaining the growing trend of NOx emissions^12^. Javed et al. explored the vibration characteristics of ZnO nano-additive biodiesel blends along with hydrogen. They developed an ANN model to forecast RMS velocity for avoiding strenuous experimentation, and these predictions were matched with experimental results as evidenced by the regression values of 0.97185, 0.98574 & 0.96913 with horizontal, vertical & axial directions correspondingly. They determined that B30 and B20 fuels (with 40 ppm ZnO) and hydrogen (flow rates of 0.5, 1.0, and 1.5 l/min) were the best fuels with the least vibrations^13^. Khandal et al. characterised the influence of FIT, EGR, and hydrogen flow rate, as well as honge and cotton biodiesel, on the engine characteristics of DF engines. They concluded that 15% of EGR shows reduced BTE (by 23–24%), higher smoke (by 28%), hydrocarbon (38–40%), and CO (31–38%) with biodiesel fuels at 80% load condition. Further, NO_x_ emissions diminished by 26–28% at 80% load equated to the CI mode^14^. Nag et al. conducted engine tests in dual fuel mode (hydrogen and diesel) to investigate the combustion analysis and vibrations of a diesel engine. The hydrogen induction with diesel reduces the noise and vibration levels, and it is more beneficial at low and average loads^15^. Sivamurugan et al. identified the influence of mustard biodiesel along with methane on the emission output of a research diesel engine. The utmost reduction of HC and CO pollutants was identified for biodiesel-methane (flow rate of 5 lpm) equated to neat biodiesel. Higher NOx emissions were found with the methane-biodiesel fuel sample at all flow rates^16^. Jaikumar et al. evaluated the noise and vibration levels of the CI engine when it was running on hydrogen and biodiesel. They conducted experiments for the B20 fuel with hydrogen induction at various flow rates (5, 10, 15 lpm), and vibration, noise levels were also measured. Compared to other test fuels, they found that NSOME mixes reduced vibration levels, while increased hydrogen flow rates further reduced noise and vibration levels^17^. Nayak et al. illustrate the production of producer gas and biogas for operating a double-cylinder dual-fuel diesel engine. When using dual fuel mode instead of diesel mode, the tailpipe pollutants, including NO and smoke opacity, were better managed. They reported that BTE was reduced by 2.47 and 1.67% for producer-gas and biogas along with diesel fuel compared diesel mode. By using biogas rather than producer gas, the emissions such as CO and NO were optimized, but compared to biogas, the producer gas produced better outcomes in terms of HC and smoke pollutants^18^.

In the last decade, many researchers have attempted to use various types of nanoparticles mixed in biodiesel and diesel fuels to expand the combustion ability of a diesel engine, as nanoparticles behaves as a catalyst in combustion process and significantly reduce emissions. Basha and Anand were investigated how distributing carbon nanotubes (CNTs) with particle sizes of 50 nm into diesel emulsion fuel affects diesel engine performance. They found a 10% reduction in BSFC, a 5% and 4% increase in CP and HRR, and a significant reduction in exhaust gases such as NOx, CO, UHC, and soot emissions^19^. Heydari et al. evaluated the impact of CNTs combined with diesel fuel on CI engine performance. According to their research, utilising B2E4C60 fuel enhanced torque by 15.52% to diesel fuel. Furthermore, SFC and EGT decreased marginally^20^. El-seesy et al. achieved the highest increase in BTE by 16% and a 15% decrease in BSFC with the MWCNTs-B20 blended fuel (dose level of 50 mg/l) as compared to the JB20D. The 20 mg/l dose level of MWCNTs resulted in 35%, 50%, and 60% reductions in NOx, CO, and UHC emissions, respectively^21^. Praveen et al. investigated the combined effect of MWCNT dispersion and EGR on diesel engines using C. Inophyllum biodiesel blends. According to the findings, adding MWCNTs and MWCNTs + EGR to B20 gasoline increases BTE by 7.6% and 2.26%, respectively. The CO, HC, and smoke pollutants were dramatically reduced by 1.42, 7.4, and 5.6% when the B20MWCNT40 fuel was compared to the B20 fuel sample^22^. Praveen et al. also investigated engine performance using TiO_2_ nanoparticles in a C. Inophyllum biodiesel blend. Their studies show that engine performance improves, and pollution levels decrease^23^. Ameer et al. shown that adding ZnO nanoparticles to WPO20 (waste plastic oil-diesel blend) dramatically reduces exhaust pollutants while improving diesel engine performance. Concurrently, a 2.47% increase in brake thermal efficiency (BTE) and a drop in brake specific fuel consumption (BSFC) were noted. These results show that ZnO nanoparticles are an effective additive for improving the emission, combustion, and performance properties of WPO20 fuel blends in diesel engines^38^. Ameer et al. found that a CFME20 mix (20% cashew nutshell biodiesel in diesel) reduced smoke, unburned hydrocarbons (UHC), and carbon monoxide (CO) emissions. However, there was a minor rise in NOx and a marginal drop in brake thermal efficiency (BTE). To improve the blend’s properties, magnetite (Fe3O4) NPs were mixed into CFME20 gasoline to create a nano-additive fuel. This adjustment resulted in a significant reduction in exhaust emissions, particularly NOx, as well as a modest improvement in BTE. Furthermore, the use of hydrogen during nano-additive pilot fuel burning considerably reduced smoke, CO, and UHC emissions while enhancing BTE. Although NOx emissions rose with increasing hydrogen flow rates, the increase was negligible at 10 LPM^39^. Suhel et al. discovered that adding ferrous ferric oxide (Fe3O4) nanoparticles of 100 ppm to biodiesel blends improves combustion with reduced harmful engine emissions. Experimental examination of the B20FFO100 blend (20% chicken fat methyl ester with 100 ppm Fe₃O₄ nanoparticles) indicated considerable improvements: BTE increased by 4.84%, while BSFC decreased by 10.44%, BSEC decreased by 9.44%, and EGT fell by 19.47%. In terms of emissions, CO levels decreased by 53.22%, UHC by 21.73%, NOx by 15.39%, and smoke opacity by 14.73%^40^. Norwazan et al. explored the use of CaO-TiO₂ nanoparticles as a catalyst in the synthesis of waste cooking oil (WCO) biodiesel is a viable alternative that eliminates the need for biodiesel washing, simplifying the process and lowering production costs. Furthermore, the catalyst is reusable, making it more economically viable. The CaO component of the catalyst was generated from waste cockle and sea snail shells, providing a low-cost and environmentally friendly source for heterogeneous catalysis^41^. Suhel et al. found that adding 100 ppm of magnetite (Fe₃O₄) nanoparticles to a CFB30 mix (30% chicken fat biodiesel with diesel) considerably improved diesel engine performance and emission characteristics. The use of nanoparticles resulted in a 7.49% gain in brake thermal efficiency (BTE), a 22.86% reduction in brake specific fuel consumption (BSFC), and a 15.72% reduction in nitrogen oxides (NOx). Furthermore, emissions of smoke, unburned hydrocarbons (UHC), and carbon monoxide (CO) were reduced by 15.64%, 22.27%, and 0.119% (vol.), respectively, when compared to the clean CFB30 blend. The subsequent hydrogen induction at a flow rate of 10 LPM increased BTE by 5.08% while decreasing smoke by 11.57%, CO by 0.037% (vol.), BSFC by 14.17% and UHC by 14.7%. Although hydrogen addition resulted in a modest rise in NOx emissions, overall engine performance improved significantly. Based on the experimental results, the combination of magnetite nanoparticles and 10 LPM hydrogen induction with CFB30 fuel can be deemed a viable technique for improving diesel engine performance and lowering emissions^42^. Bisheswar et al. explains the supercritical process (SCP) which enables high-quality biodiesel production from diverse feedstocks, even with high water or FFA content, using solvents like methyl acetate or dimethyl carbonate. However, its major drawbacks include high operational costs and potential thermal degradation. Superheated vapor (SHV) technology offers an emerging alternative, though comparative studies remain limited. Future work should focus on optimizing reactor design, improving fuel stability, and reducing production costs^43^. Adamczak et al. reported biodiesel synthesis was predominantly achieved via chemical catalysis, though enzymatic and microbial approaches are gaining attention for future petroleum diesel alternatives. Lipases are widely used to produce fatty acid methyl esters (FAME), while engineered microorganisms offer promising biosynthetic pathways. Although enzymatic methods face cost-related challenges, recent advancements have enabled their industrial application. These biotechnological processes are particularly suitable for low-quality feedstock like waste oils, which are unsuitable for conventional chemical methods^44^. Muhammad et al. reviewed recent advances in biodiesel production technologies, emphasizing solid catalysts and non-catalytic supercritical processes. It compares conventional methods—such as homogeneous, heterogeneous, and enzyme catalysis, as well as microwave and ultrasonic-assisted techniques—with supercritical synthesis. The supercritical method offers notable advantages, achieving up to 71.6% energy savings. Key process parameters affecting yield are discussed, alongside an economic analysis and insights for future research directions^45^. The researchers are now focusing on incorporating the machine learning techniques in the behaviors study of automotive engines^48^.

The previously reviewed literature concludes that hydrogen induction in the input manifold and the incorporation of nano particles into biodiesel and diesel fuels improve combustion efficiency and reduce emissions in a diesel engine. However, there is little research on the comparative effects of nanoparticles and hydrogen enrichment on biodiesel blended fuels. Lemongrass biodiesel, with its renewable origin and favourable combustion qualities, offers a possible alternative to conventional diesel. The use of nanoparticles, notably due to their catalytic behaviour and huge surface area, has shown promise in improving fuel-air mixing, combustion behaviour, and emissions reduction. Furthermore, when blended with liquid fuels, hydrogen, with its fast flame speed and negligible carbon content, has the potential to dramatically improve thermal efficiency and reduce carbon emissions. Despite various studies on biodiesel-nanoparticle and hydrogen dual fuel technology, there is a paucity of comprehensive research on the combined effects of nano-enhanced lemongrass biodiesel and hydrogen premixing, particularly in terms of synergistic effects on engine performance and emissions. The current study is unique in that it conducts a systematic investigation of multi-walled carbon nanotubes (MWCNTs) as a nano-additive in lemongrass biodiesel, in conjunction with hydrogen premixing, to determine their combined effect on combustion efficiency, fuel economy, and emission reduction in a CI engine. Thus, the current study investigates the comparative influence of MWCNTs (40 ppm) and hydrogen addition (5 lpm) in lemongrass-diesel blended (LG20) fuel to evaluate the combustion (cylinder pressure, heat release rate, and ignition delay), performance (BTE, BSFC, EGT), and emission characteristics of a diesel engine (CO, HC, CO2, NOx, and smoke opacity).

## Materials and methods

### Production of Lemongrass biodiesel

Lemongrass (Cymbopogon Citratus) plant was cultivated in tropical and sub-tropical parts of Southeast Asia, Africa, and especially in India, where it is cultivated in all southern states. Lemongrass plant has dense bunches and thrives in moist and loose types of soil. Its leaves appeared in green color with sharp edges, and the harvesting period is about 80 days, which depends on the fertility of the soil. Lemongrass oil can be extracted by using the hydrocarbon solvent (n-hexane) extraction method from the leaves and stalks of the plant. Lemongrass oil is a non-edible oil and potential biodiesel feedstock, was extracted using the hydrocarbon solvent n-hexane to ensure high lipid recovery and purity. The solvent extraction method was selected due to n-hexane’s non-polar nature and low boiling point, which facilitates efficient oil solubility and recovery under mild operating conditions. Pre-treated lemongrass was subjected to Soxhlet-based extraction, achieving superior oil yields compared to conventional mechanical pressing. The recovered oil exhibited improved fuel-relevant properties post-transesterification, validating its suitability for biodiesel production. Despite concerns over solvent toxicity and flammability, the process’s advantages such as higher extraction efficiency, selective lipid recovery, and ease of solvent recycling supports the continued use of n-hexane in biodiesel research. The leaves were freshly cut (up to 10 cm from the root), generally in the morning of the day, slashed into small parts, then they dried in 4–5 days at room temperature and protected from sunlight. A total of 300 g of dried lemongrass samples were placed in a flat-bottomed flask, and 1 l of n-hexane was added as the solvent. The mixture was left undisturbed for 48 h to facilitate oil extraction. After the extraction period, the solution was carefully transferred to a separate beaker for further processing. The bottom layer, containing the ethanol-extracted oil, was carefully separated and transferred to a clean beaker. Ethanol was added during the extraction of lemongrass oil to enhance the solubility of active compounds, reduce oil viscosity for easier separation, act as a mild dehydrating and preserving agent, and ultimately improve extraction efficiency and yield. Next, 250 ml of ethanol was mixed into the extracted oil, and then this mixture was stirred for 20 min, and finally transferred into a funnel for the liquid/liquid separation process to divide into two layers. The bottom layer is ethanol extraction oil, which is collected into a separate beaker, and it was then heated to approximately 90°C to evaporate the residual ethanol and obtain purified lemongrass oil and the yield varies based on the feedstock quality.

Lemongrass oil is a low viscosity oil; transesterification is essential to convert it into biodiesel suitable for CI engines. Raw lemongrass oil lacks critical fuel properties such as adequate cetane number, energy density, and stability, which are necessary for efficient and clean combustion. Transesterification improves these properties, making the fuel compatible with engine requirements and emission standards. This conversion not only improves engine compatibility but also ensures cleaner combustion and reduced emissions, making lemongrass-derived biodiesel a viable alternative fuel.

**Fig. 1:**
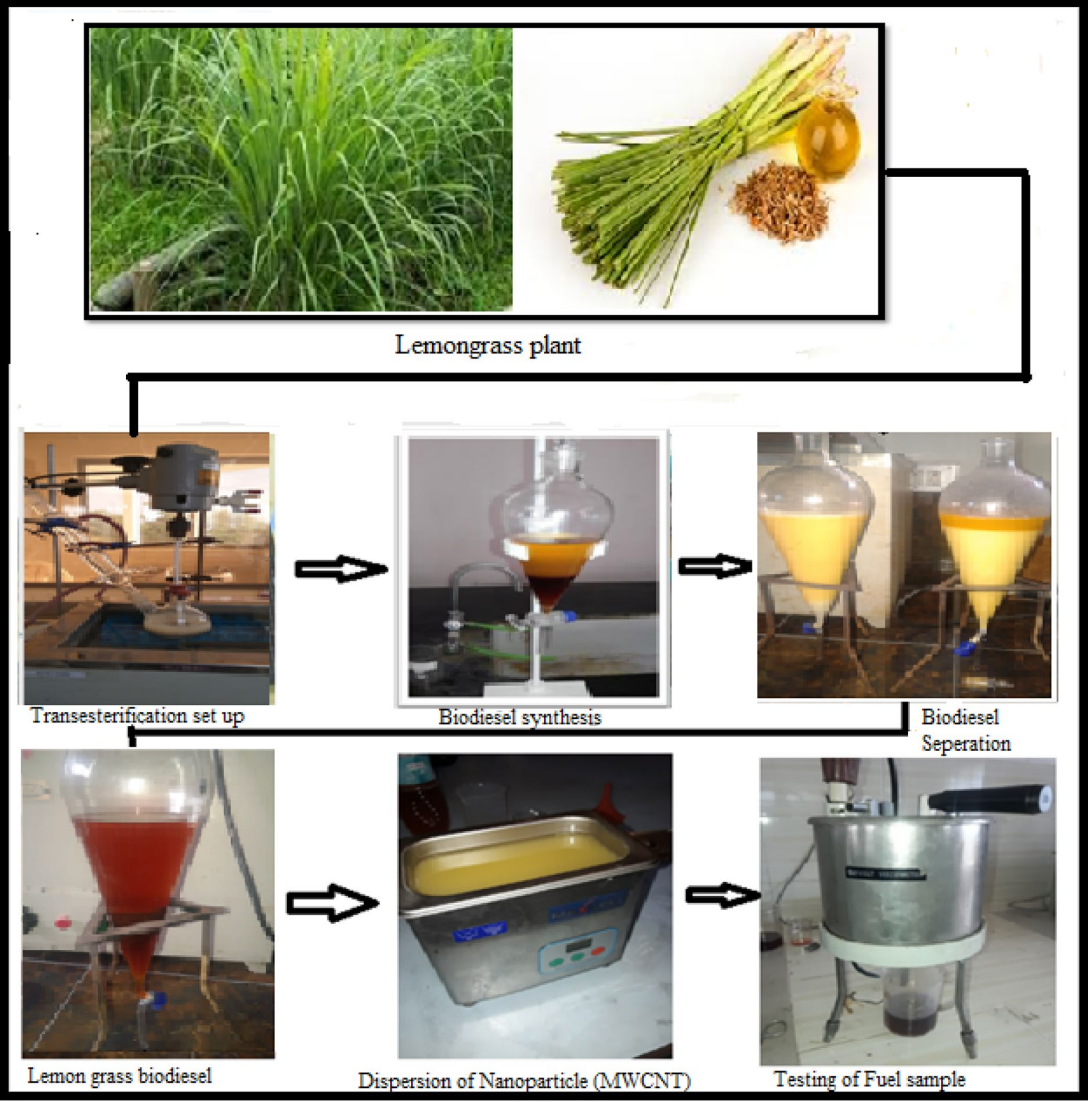
Preparation of Biodiesel, Dispersion of nanoparticle, and testing of fuel properties.

To handle the entire research, for the present study the lemon grass oil was purchased with a local vendor in Kerala Tamil Nadu, India. For The transesterification technique is used to create biodiesel from lemongrass (LG) oil with the help of a catalyst. The LG biodiesel is made with methanol to oil ratio of 6:1 along with KOH (Potassium hydroxide) as the catalyst. Initially, lemongrass oil is put into the reactor, followed by the addition of methanol and catalyst in the desired proportion. This mixture is stirred for one hour at 600 °C. After that the mixture is then transferred to a separating funnel and allowed to settle for six hours. After the separation was complete, the bottom layer, glycerol, was removed from the funnel, while the top layer, biodiesel, was collected and rinsed with water to remove any excess alcohol and catalyst from it. Finally, it is heated to separate the water particles from the biodiesel. Figure 1 shows the extraction of biodiesel and the composition of nano fuel. Tables 1 and 2 indicate the comparative properties of the fuel samples.

**Table 1:** Fuel sample properties.

Property	Diesel (C_8_-C_20_)	LGBD(B20)	Hydrogen (H_2_)	Lemongrass oil
Density (kg/m^3^)	840	825	0.09	940
Kinematic viscosity (cSt) 40 °C	3.35	3.90	-----	5.24
Cetane Number	51	51	-----	51
Flash point	68	65	-----	94
Molecular Weight (g/mol	210	-------	2.016	------
Calorific value (MJ/kg)	43.5	38.55	120	37.40
Auto Ignition temperature	-----	------	310	570

**Table 2:** Distinguish between a fuel’s combustion and physical properties of various fuels^38^.

Properties	Lemon grass biodiesel	Hydrogen	CNG	Ethanol	Petrol	Diesel
Formula	--	H_2_	CH_4_	C_2_H_5_OH	C_4_–C_12_	C_8_–C_20_
Density (1 atm, 20 °C (kg/m^3^))	853	0.08	0.65	809.9	720–780	820–860
Motor octane number	-	130	105	89.7	95–97	–
Flammability limits in air (%Vol.)	-	4–74.5	5.3–15	3–19	1.4–7.6	0.6–5.5
Stoichiometric ratio (kg/kg)	-	34.3	17.2	9	14.7	14.5
Auto ignition temperature (°C)	-	572	540	363	257	254
Min. ignition energy (MJ)	-	0.02	0.29	0.23	0.23	–
Adiabatic flame temperature (K)	-	2400	2320	2193	2300	2200
Maximum flame speed (m/s)	-	3.5	0.42	0.61	0.5	0.3
Min. quenching diameter (mm)	-	0.9	3.53	2.97	2.97	–
Lower heating value (MJ/kg)	37.40	120	49.99	26.7	43	42.5

### FTIR of Lemongrass oil

**Fig. 2:**
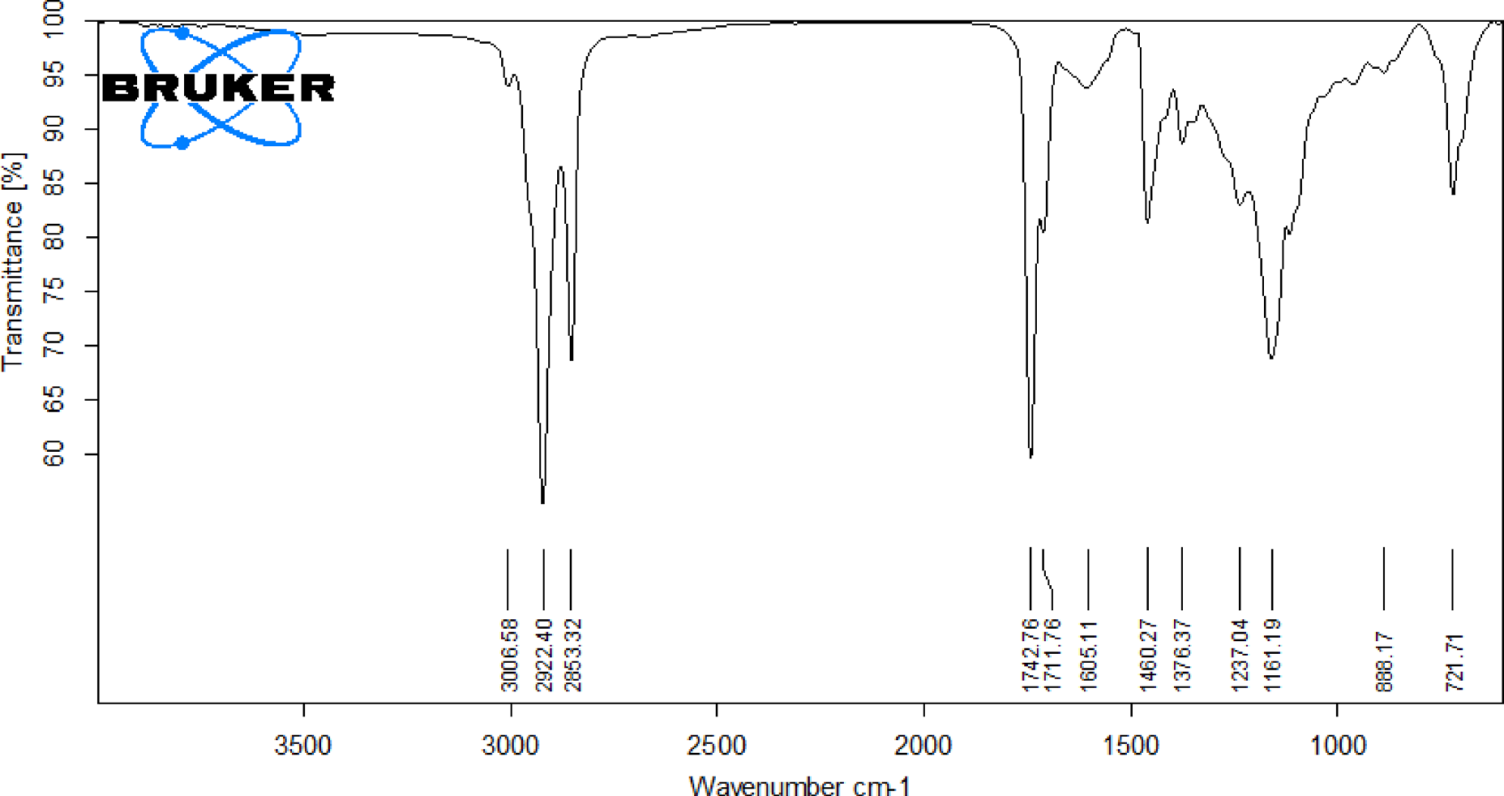
FTIR report of C. Inophyllum raw (Lemon Grass) oil.

**Fig. 3:**
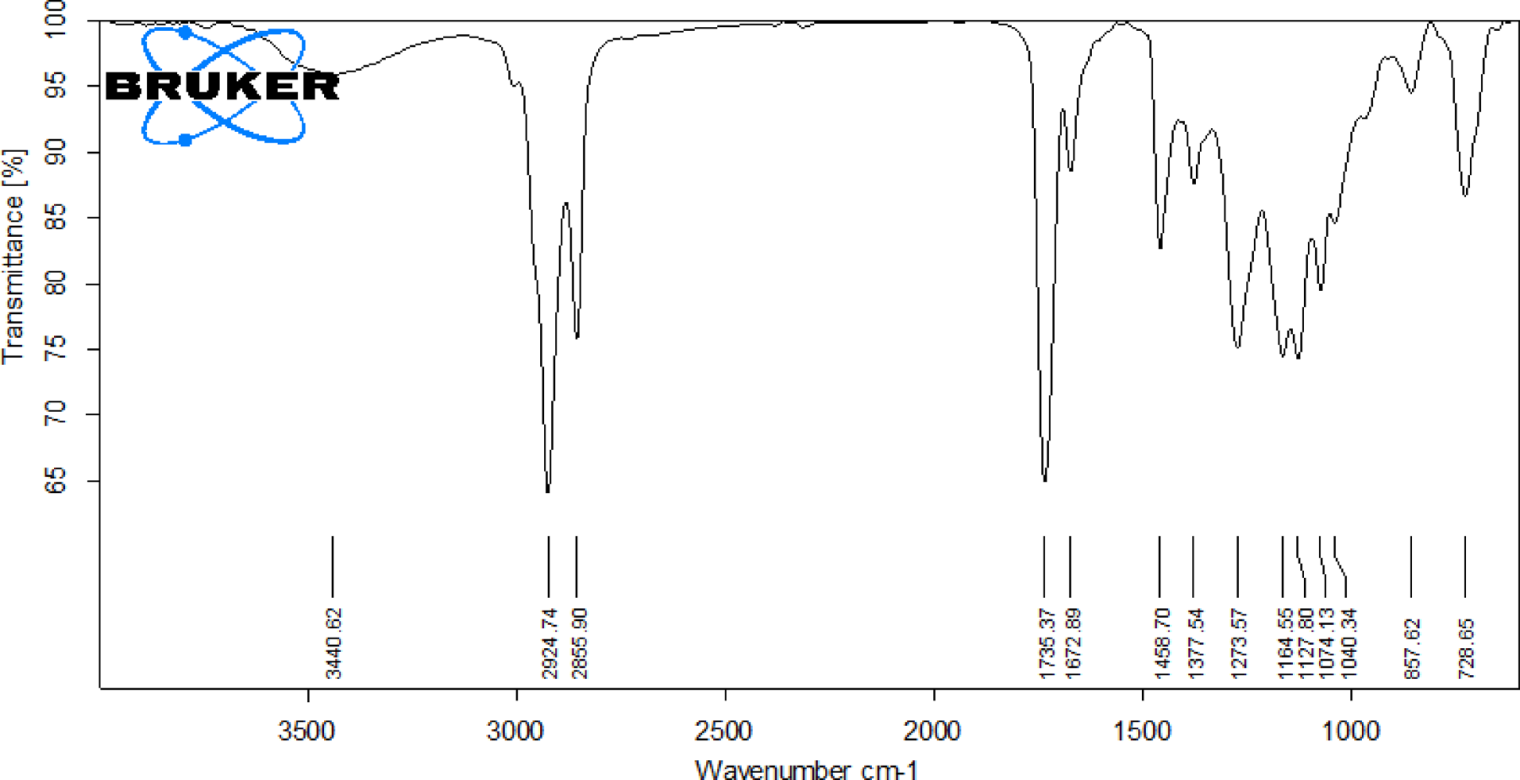
FTIR report of Lemongrass Cymbopogon Citratus (Lemon grass) biodiesel.

The lemongrass biodiesel has been examined by FTIR with a Bruker spectroscopy model in the 4000 –400 cm^− 1^ range to identify functional groups and bands corresponding to various vibrations, as illustrated in the Figure. The stretching vibrations of C-H appear at 3440.62 cm^− 1^. The deformation of the methyl and methylene groups led the band to develop at 2924.74 and 2855.90 cm^− 1^, respectively. The methoxy carbonyl group in lemongrass biodiesel appeared in the range of 1735.37 to 1672.89 cm^− 1^. The peak at 1074.13 corresponds to the peak of (C-O) ester, and the additional two crests at 1164.55 and 1127.80 cm^− 1^ specify the translation of oil into biodiesel. The absorption peaks appeared at 857.62 and 728.65 cm^− 1^ signify the formation of CH_2_ groups. Table 3 depicts the Gas Chromatographic Molecular Weights (MW) and Retention Times (RT) of Fatty Acid Methyl Esters (FAME) from lemongrass Triglycerides. Figure 2 represents the FTIR investigation of C. Inophyllum raw oil, and Fig. 3 shows the FTIR Analysis of Lemongrass Cymbopogon Citratus (Lemon grass) biodiesel.

**Table 3:** Molecular weights (MW) and gas chromatographic retention times (RT) of fatty acid Methyl esters (FAME) from lemon grass Triglycerides.

Compound in Fatty acids	FAME	
	RT (min)	M.W
C16:0	15.2	274
C18:1	17.23	296
C20:1	18.9	325
C22:1	21.6	348
C24:1	23.20	378

### Characterization of MWCNT

**Fig. 4:**
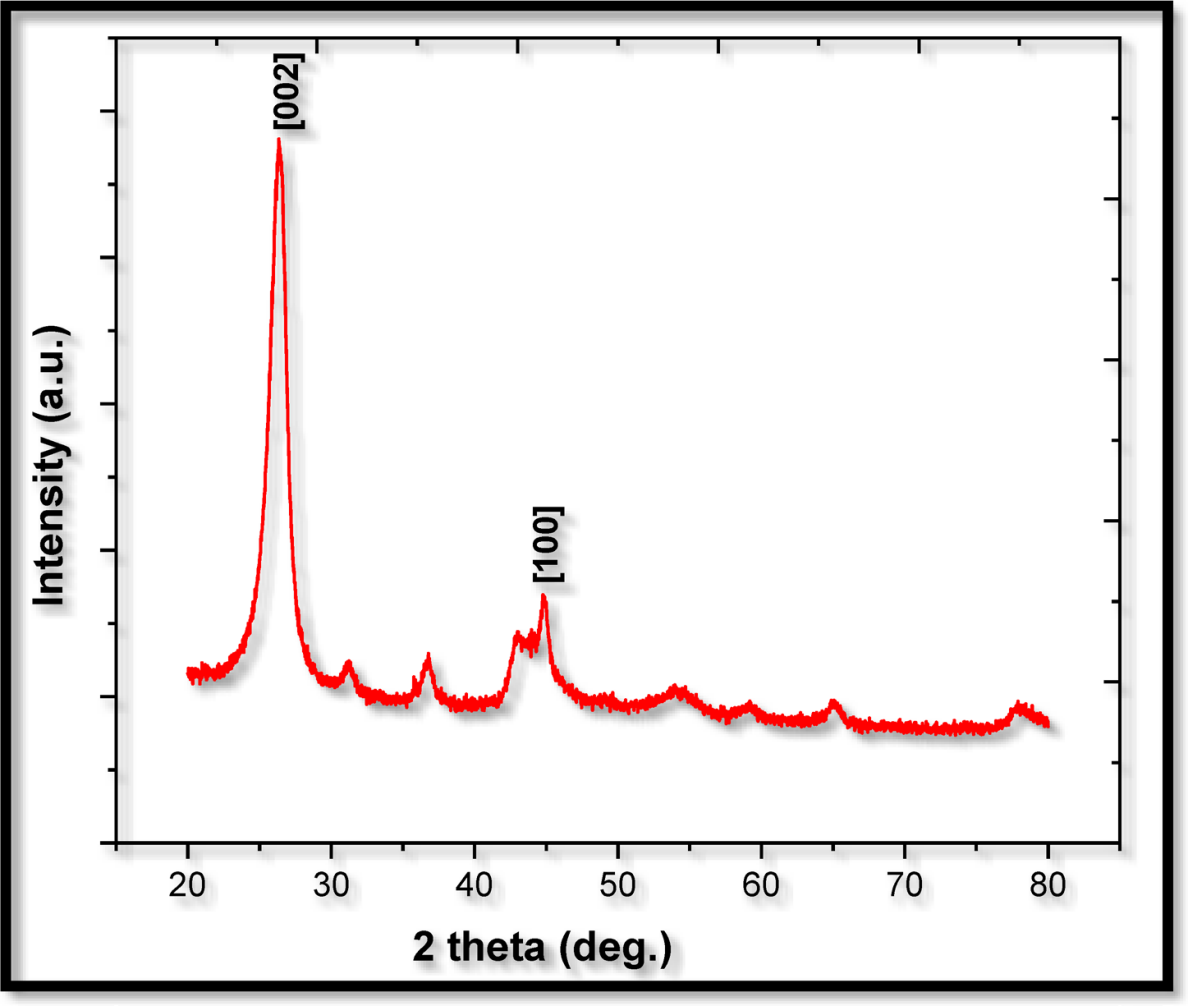
XRD of MWCNT.

**Fig. 5:**
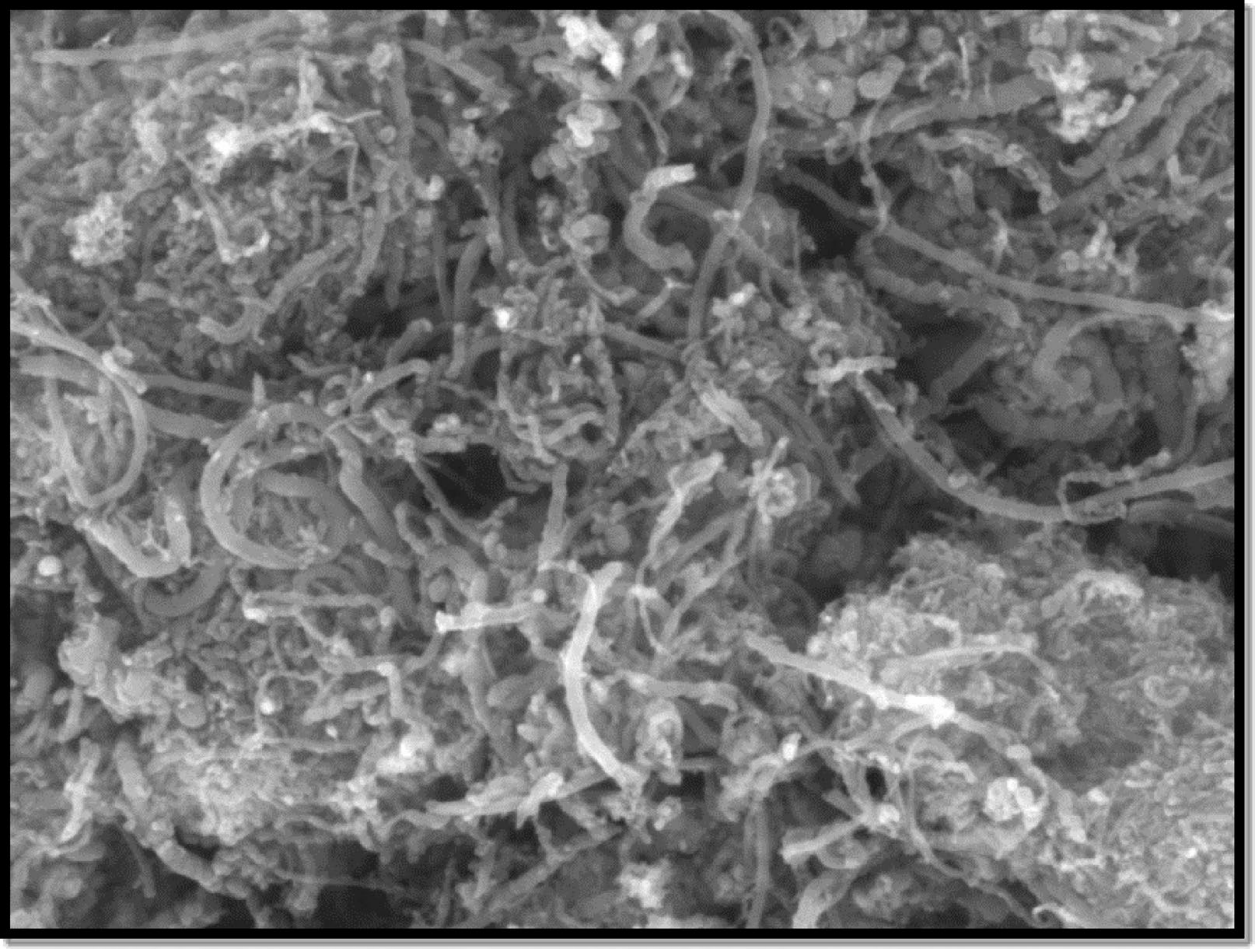
SEM picture of MWCNTs.

**Fig. 6:**
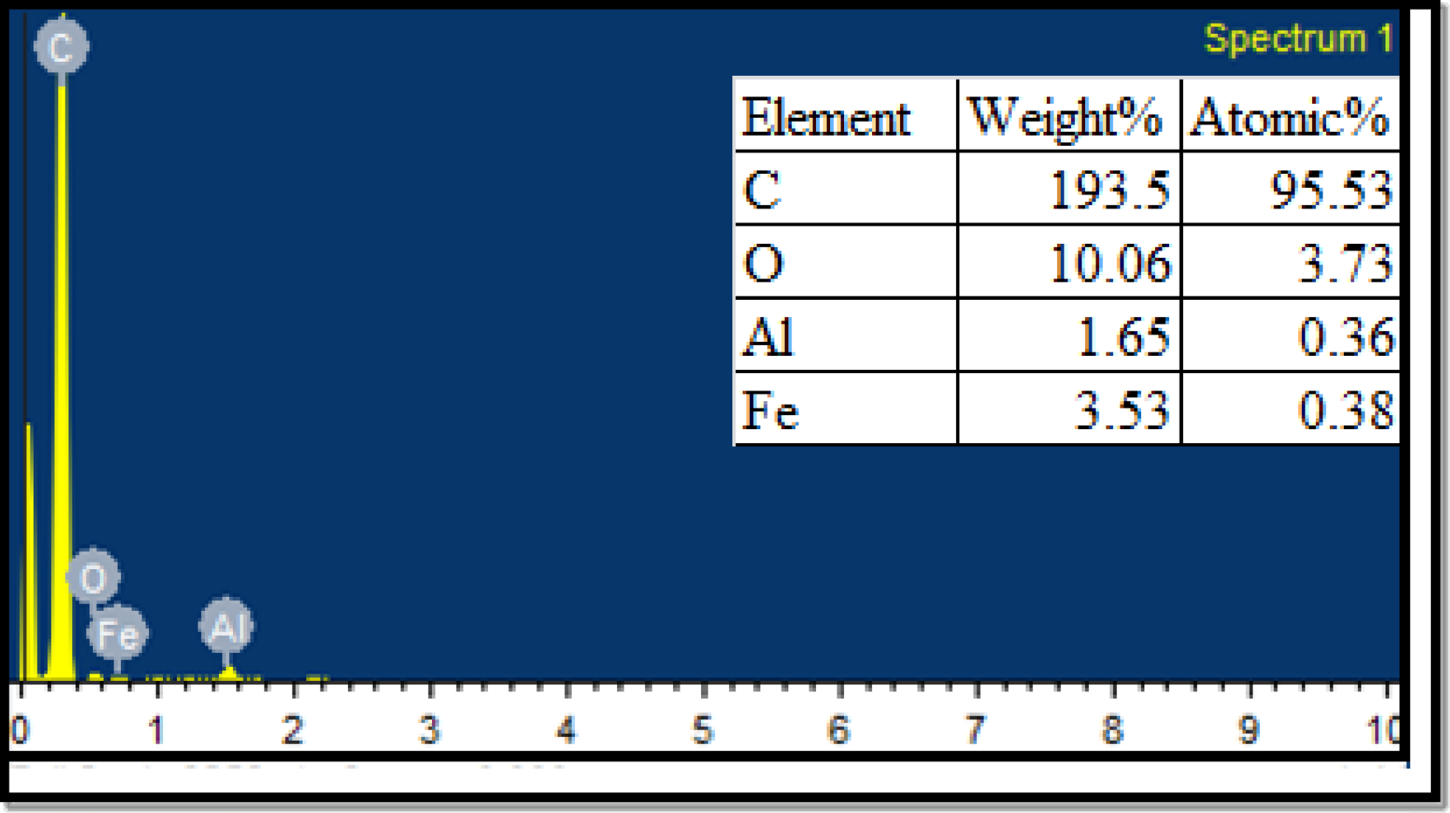
EDAX of MWCNT.

Nano Wings Pvt, Ltd., based in Khammam, Telangana, India, was supplied the multiwalled carbon nanotubes (MWCNT). Table 4 provides information on the nanoparticles. The multiwalled carbon nanotubes were characterised using energy dispersive spectroscopy (EDS), scanning electron microscopy (SEM), and X-ray diffraction (XRD) methods. Figure 4 illustrates the crystalline structure of multiwalled carbon nanotubes that was studied using the X-ray diffractometer (XRD). For multiwalled carbon nanotubes, the different crests in the XRD pattern were found at (002), (100), (004), and (110). The XRD pattern’s (002) peak indicates that carbon nanotubes are multiwalled (Mary Anjalin, 2014). Figure 5 displays the MWCNTs SEM image. Using SEM analysis, the surface morphology and measurements of multiwalled carbon nanotubes were determined. Employing the SEM technique, it was determined that the multiwalled carbon nanotubes’ tube length was, and external diameter are 1–10 μm and 10–30 nm, respectively. Multiwalled carbon nanotubes were analyzed to determine their elemental composition using energy-dispersive spectroscopy. Figure 6 EDAX spectrum for MWCNTs verifies the sample’s C, O, Fe, and Al composition. Table 4 displays the details of MWCNT. Using an ultrasonicator running at 120 W, 40 kHz for an hour, the MWCNTs were dispersed across the biodiesel-diesel blend (B20) in a mass fraction of 40 ppm. Table 5 displays the results of the ASTM-standard measurements made on the LG20 and LG20MWCNT40 fuel samples.

**Table 4:** Details of MWCNT.

Particulars	Specifications
Name	Multi wall carbon Nano Tube (MWCNT)
Merchant	Nano Wings Pvt. Ltd, Khammam, TS, India
Size	20–30 nm
Purity	> 90%
Specific surface area	450 m^2^/g
Length	0–10 μm
Appearance	Block

**Table 5:** Distinguish between a tested fuel’s physical and combustion properties^38,46,47^.

Properties	ASTM standards	Lemon grass oil	Lemon grass biodiesel (B20)	Lemon grass biodiesel (B20) + 40ppm (MWCNT)
Kinematic viscosity, cSt at 40 °C	ASTM D1298	5.24	3.90	3.28
Density (1 atm, 20 °C (kg/m^3^))	ASTM D445	890	853	854.4
Flash point, (°C)	ASTM D92	94	65	68
Fire point, ⁰C	ASTM D92	100	72	75
Calorific value MJ/kg	ASTM D6751	37.40	38.65	39.26
Cetane number	ASTM D613	51	51	50.6

### Hydrogen

Hydrogen is a promising alternative fuel that can power a variety of vehicles and machines, such as cars, trucks, buses, and trains. One of its biggest advantages is that it burns cleanly producing only heat and water when combusted. This makes hydrogen an attractive option for cutting greenhouse gas emissions and improving air quality in cities. However, there are challenges to using hydrogen as a fuel. A major hurdle is the high cost involved in producing, storing, and transporting it. Although new technologies are being developed to make these processes more efficient, current methods remain expensive and energy demanding. Additionally, the infrastructure needed to support hydrogen fuelling stations is complex and costly, since hydrogen requires specialized storage and handling equipment unlike conventional fuels. Building a widespread network of hydrogen stations would require significant investment. Despite these challenges, hydrogen continues to be seen as a viable fuel option. As technology advances and production scales up, the costs associated with hydrogen are expected to decrease, making it a more practical choice for widespread adoption in the future.

## Experimental setup and procedure

**Fig. 7:**
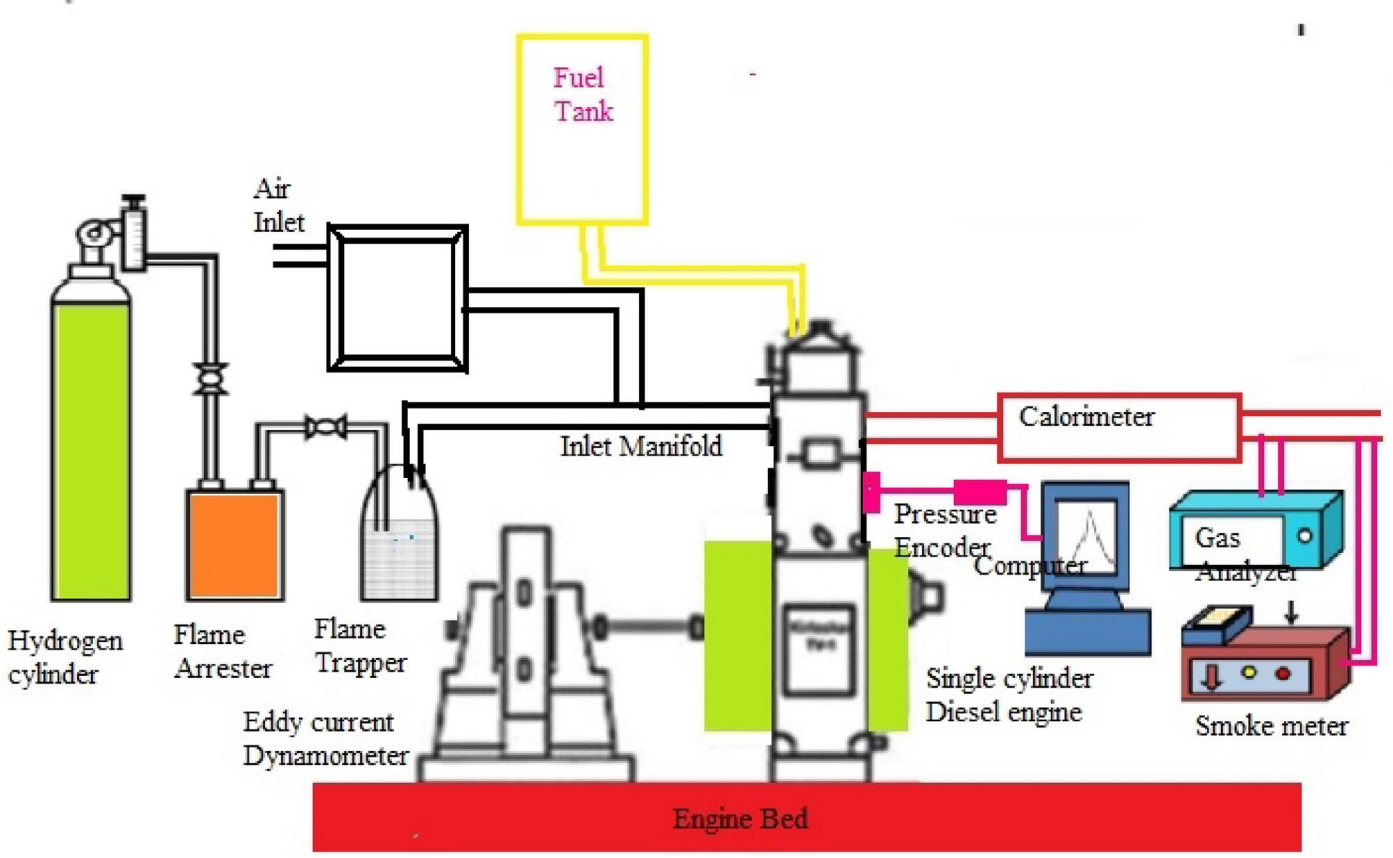
Schematic diagram of the test engine with gas analyzer and smoke meter.

**Fig. 8:**
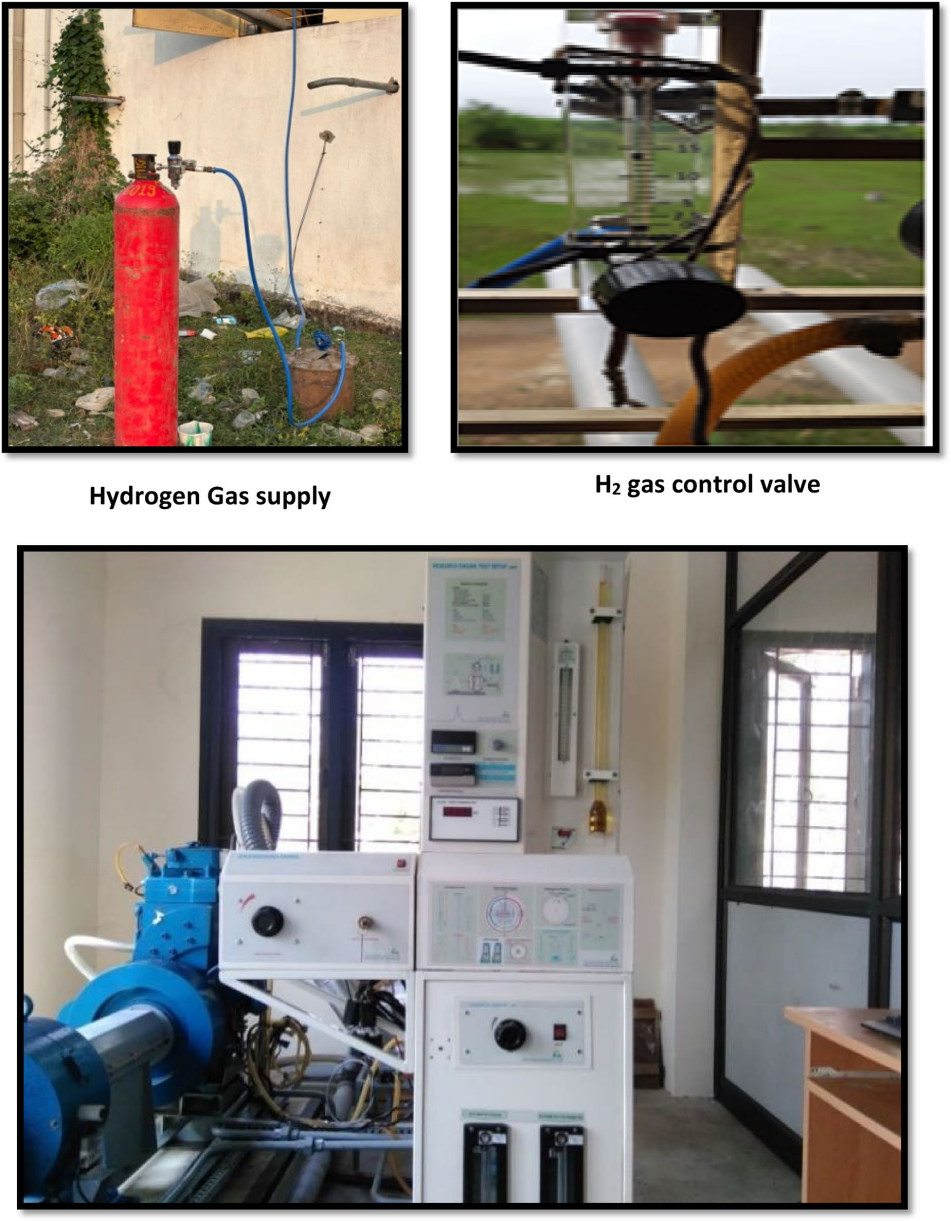
Photographic view of Test Engine setup.

Initially, hydrogen gas was stored in high-pressure cylinders, with pressure regulators and flame arresters used to assure safe and controlled distribution of the gas. The pressure was kept at the proper level with the help of the pressure regulator. The large flammability limitations and high reactivity of hydrogen created a significant risk of backfiring^27^. Installing flame arrestors eliminates these disadvantages. The hydrogen flow rate was measured with suitable flow meters. Eddy current dynamometers are useful for determining the torque produced by the engine and serving as load characteristics. A flue gas analyser was used to test various elements in engine exhaust during combustion. The ECU uses a computer to precisely manage the EFI system. Figure 7 displays the test setting for the electronic assisted fuel injection CI engine used in this study. Figure 8 depicts the test engine, a 4-stroke, mono-cylinder, water-cooled diesel engine built by Kirloskar. The engine specifications are shown in Table 6. This engine is designed to operate on two different fuels. Initially, it was powered by diesel and biodiesel blends (LG20), with fuel samples containing MWCNTs. To test engine power and emission parameters, hydrogen gas was combined with clean air and pumped into an engine’s intake manifold containing diesel, LG20, and LG20 + MWCNT fuel samples at a constant speed of 1500 rpm. For safety, a water-filled flame trap and a flame arrester were installed in the gaseous fuel (hydrogen) entry. The fuel flow is regulated by the flow control valve. The fuel injection pressure was increased to 200 bar by extending the injector’s nozzle while maintaining a fuel injection angle of 23^0^ bTDC. To apply load to the engine from 0 to 100%, an eddy current air-cooled dynamometer is used. A burette with the proper valves was mounted in the panel to gauge the tested fuel samples, and a timer was used to record the fuel flow rate. Exhaust gas temperatures were measured using K-type thermocouples. A piezoelectric transducer and a crank angle encoder were installed on the cylinder head to properly detect crank angle and cylinder pressure respectively. Data acquisition devices were installed in an engine with a computer interface to analyse and evaluate data collected from various devices utilising engine soft software 4.0. The experiment used sample fuel of diesel, LG20, and LG20 with MWCNT and hydrogen gas at 5 lpm under all load conditions. The exhaust gases were analysed using an AVL444 gas analyser, and the smoke pollutants were measured with an AVL437 smoke meter. Table 7 shows the precision and range of each instrument employed in this test engine. Table 8 summarises the results of the experiment’s uncertainty analysis. Table 9 summarises the current investigation’s findings and earlier published investigations. The test’s overall uncertainty was estimated to be ± 1.3%^19,20^.

**Table 6:** Engine specifications.

Engine Make and Model	4-stroke TV1 Kirloskar single cylinder diesel engine
Rated brake power and speed	5.2 kW and 1500 rpm
Number of cylinders	Single
Stroke × Bore (mm)	110 × 87.5
Dynamometer	Eddy current dynamometer
Standard FIP & FIT	210 bar and 23^0^ bTDC
Cooling system	Water Cooled
Compression ratio	16.5:1

**Table 7:** Smoke meters and exhaust gas analyzers’ ranges and accuracy.

Instrument	Measuring range	Accuracy
AVL Gas analyzer		
Carbon monoxide (CO)	0–9% Vol	0.01% Vol
Oxides of Nitrogen (NOx)	0-5000 ppm	1 ppm
Hydrocarbon (HC)	0-1000 ppm	1 ppm
AVL Smoke meter		
Smoke density	0-9.99 HSU	0.01 HSU

**Table 8:** The uncertainties in the measured parameters.

Variables	Nomenclature	% UNCERTAINTY
Load	L	0.6%
Brake power	BP	0.5%
Brake Specific fuel Consumption	BSFC	0.3%
Break Thermal Efficiency	BTE	0.4%
Nitrogen Oxides	NO_X_	0.5%
Carbon monoxide	CO	0.6%
Hydrocarbon	HC	0.2%
Smoke opacity	SO	0.3%
Exhaust gas temperature	EGT	0.2%

The overall uncertainty of the experiment = ± 1.3%.

**Table 9:** Comparison of the current study to previously published studies.

Fuel Combination	Type of Engine Consider for Work & Capacity	Performance		Combustion			Pollutants				Ref
		BTE	BSFC	Cylinder pressure	HRR	Ignition delay	HC	CO	NOx	Smoke	
Diesel_CIO(B20) _H_2_	CI engine 3.5 kW	↑	↓	↑	↑	↓	↓	↓	↑	↓	^24^
Diesel-H_2_, palm B20-H_2_	CI engine 3.5 kW	↑	↓	↑	↑	↓	↓	↓	↑	↓	^25^
Diesel_H_2_	CI engine, 10.3 kW	↑		↑	↑	↓	↓	↓	↑		^26^
B5_H_2_	power generator 50 kW	↑	↑	↑	↑	---	↓	↓	↑	--↓	^27^
JME_H_2_	CI engine, 4.4 kW	↓	↓	↑	↑	↓	↓	↓	↑		^28^
Diesel_H_2__biogas	CI engine 3.5 kW	↑	↓	↑	↑	---	↓	↓	↑	↓	^29^
Diesel-H_2__LG20 + MWCT	CI engine 3.5 kW	↑	↓	↑	↑	↓	↓	↓	↑	↓	Current study

## Results and discussions

### Brake thermal efficiency (BTE)

**Fig. 9:**
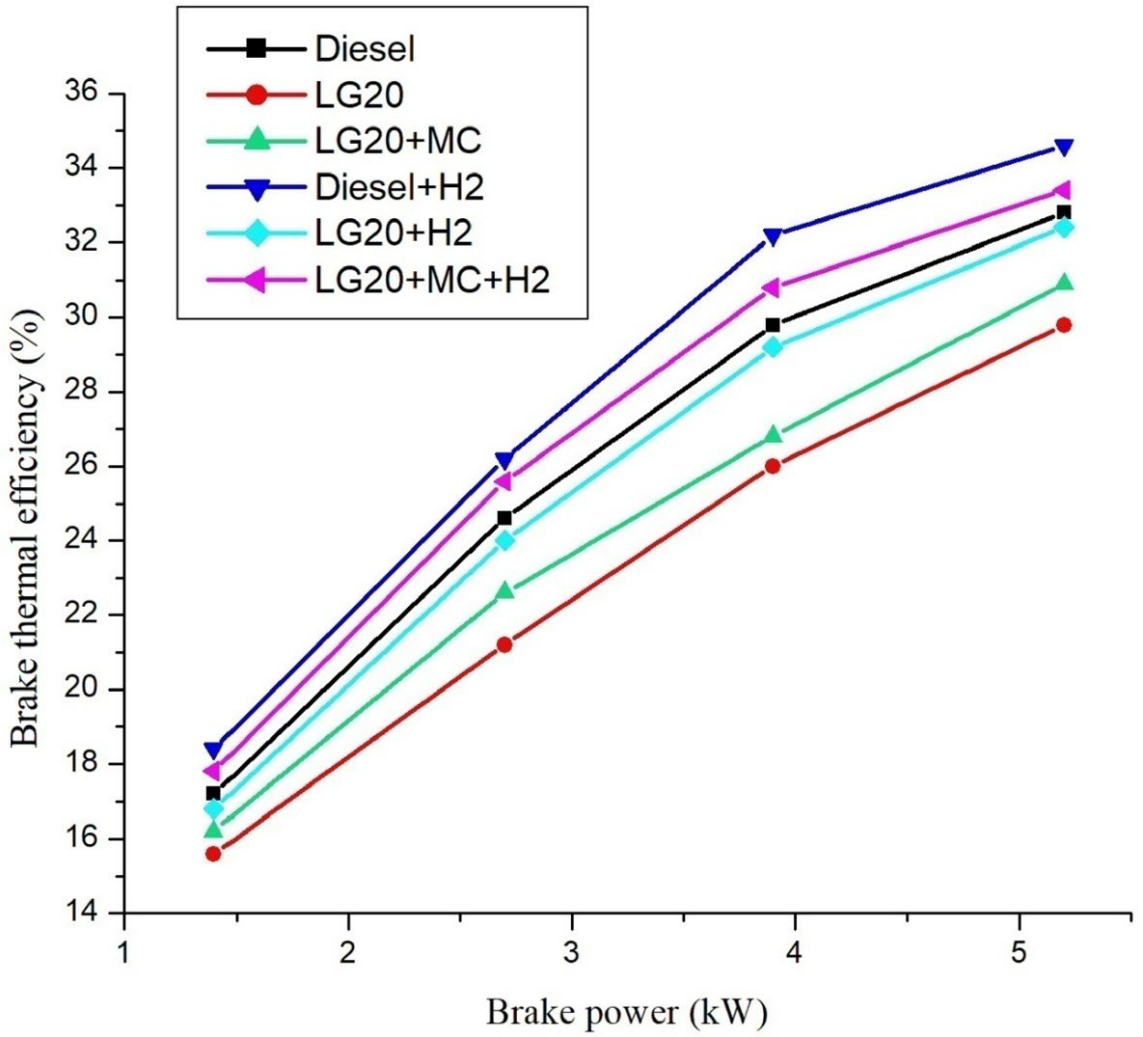
Distinctions of brake thermal efficiency and brake power.

Figure 9 depicts the variances in BTE across all test fuels. The improvement in brake thermal efficiency was attributed to an increase in engine brake power across all fuel types. The hydrogen-enriched fuels are more efficient than other fuels. The LG20 fuel shows decreased BTE ( by 10.25%) compared to the diesel, but it is compensated with the hydrogen induction to LG20 fuel due to the substantial diffusivity and flame propagation of hydrogen. The presence of MWCNTs in the LG20 fuel BTE was enhanced by 4.39% than LG20 fuel and further enhanced by 12.6% with the addition of hydrogen because of the MWCNTs and hydrogen presence, which leads to improved combustion rate^22,23^. LG20 + H_2_ + MWCNTs fuel sample exhibits higher BTE equated to remaining fuels due to the superior calorific values of diesel, H_2,_ and increased flame initiation and propagation^25,27–29^.

### Brake specific fuel consumption (BSFC)

**Fig. 10:**
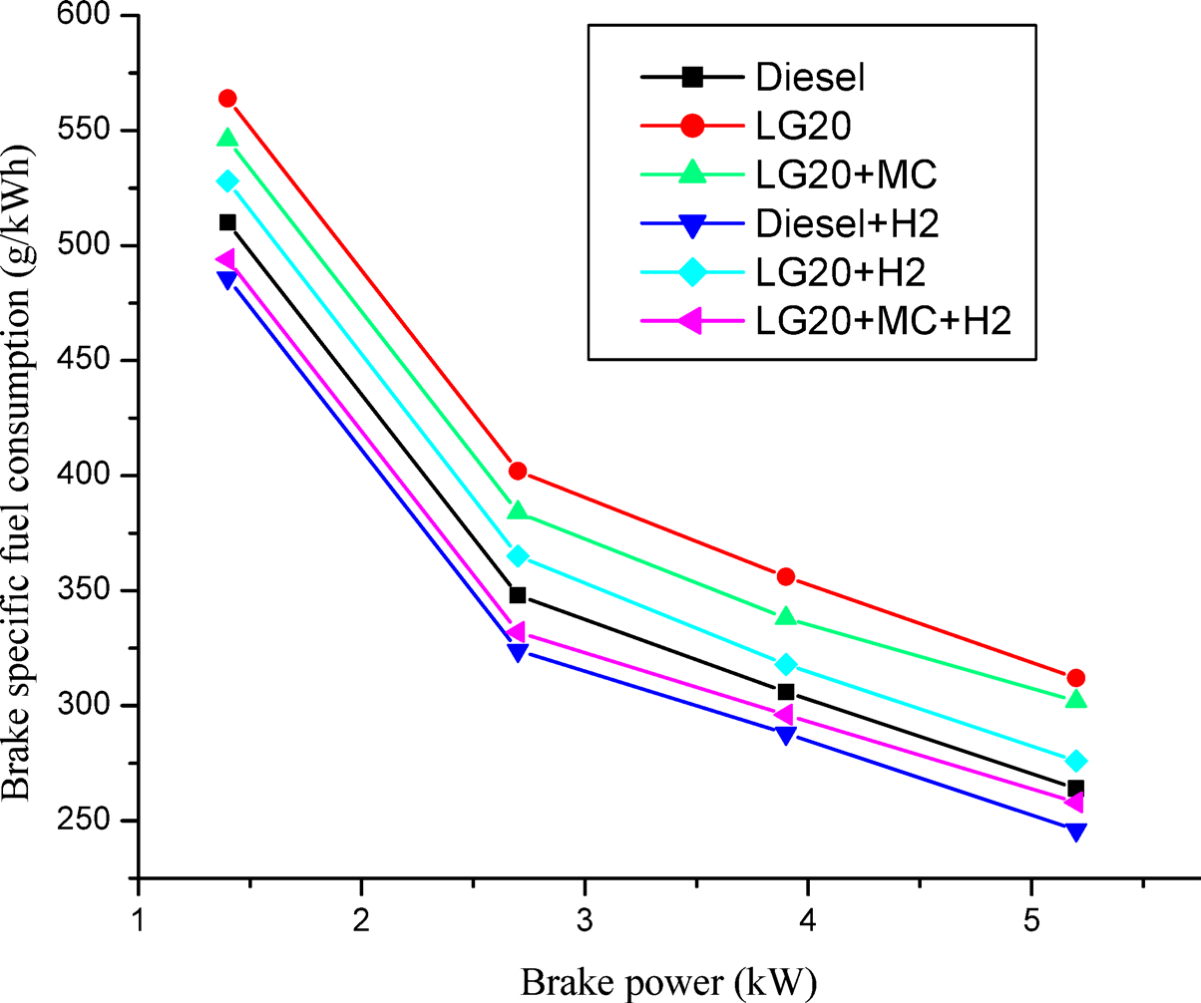
Distinctions of brake specific fuel consumption and brake power.

Figure 10 illustrates the variations in BSFC and BP for all test fuels. The LG20 fuel exhibits higher fuel consumption compared to the other fuels, which can be attributed to its higher density, greater kinematic viscosity, and lower heating value (represented in Table 1). The dispersion of MWCNTs in LG20 fuel resulted in a 3.31% reduction in brake-specific fuel consumption (BSFC), which was further reduced by 13.04% with the addition of hydrogen, compared to the LG20 fuel, due to improved fuel atomisation, and H2 gas induction causes effective fuel-air homogeneity^24,26,28^. The BSFC for LG20 + MWCNT + H2 fuel was lowered by 2.32% and 15.33% compared to diesel and LG20 fuel. Because of H2’s better calorific value, LG20 + H2 fuel consumed the least amount of fuel of any of the test fuels. At all braking power conditions, the BSFC for the LG20 + MWCNT + H2 and diesel + H2 fuels varied little^25,27–29^.

### Combustion variables

#### In cylinder pressure

**Fig. 11:**
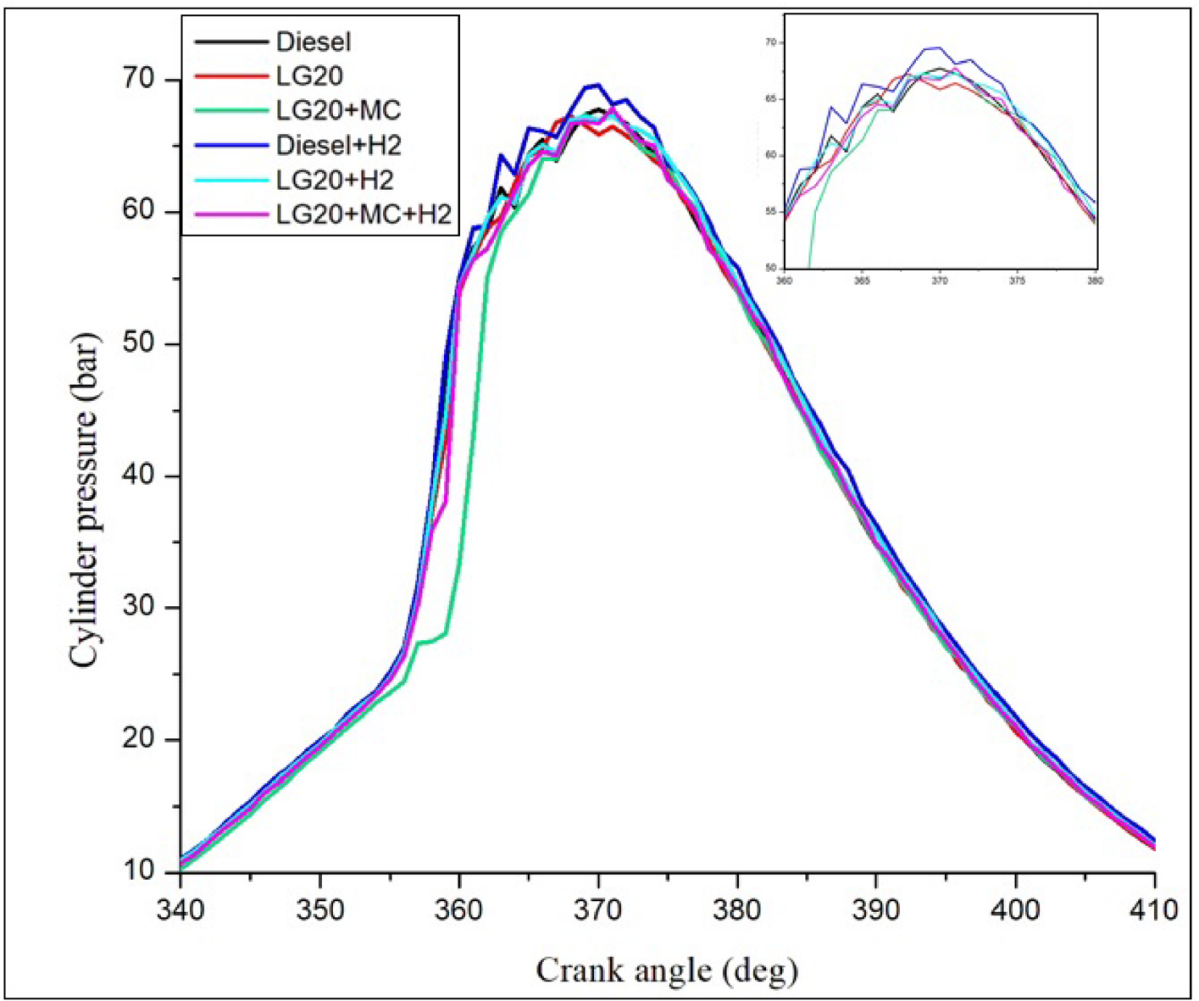
Distinctions of cylinder pressure and crank angle.

Figure 11 depicts the differences in cylinder pressure due to crank angle for all tested fuels. The peak in-cyl. Pressure was obtained for LG20 + H_2_ fuel (69.2 bar) than for other fuels at full load. The lower energy content of LG20 fuel causes a lower peak pressure (64.6 bar)than all fuel samples. Because of the better evaporation and catalytic activity, LG20 + MWCNT (65.8 bar) fuel shows maximum cylinder pressure in comparison with LG20 fuel. This combination of MWCNT and H_2_(LG20 + MWCNT + H_2_ fuel has 67.2 bar) exhibits a slight improvement in the peak pressure than LG20 + MWCNT (65.8 bar) and LG20 + H_2_ (66.4 bar) fuel samples due to a greater combustion rate and proper mixing of hydrogen gas in the atomized form of LG20 fuel. Hydrogen enrichment in LG20 fuel improves the flame front propagation^25,27–29^.

#### Heat release rate (HRR)

**Fig. 12:**
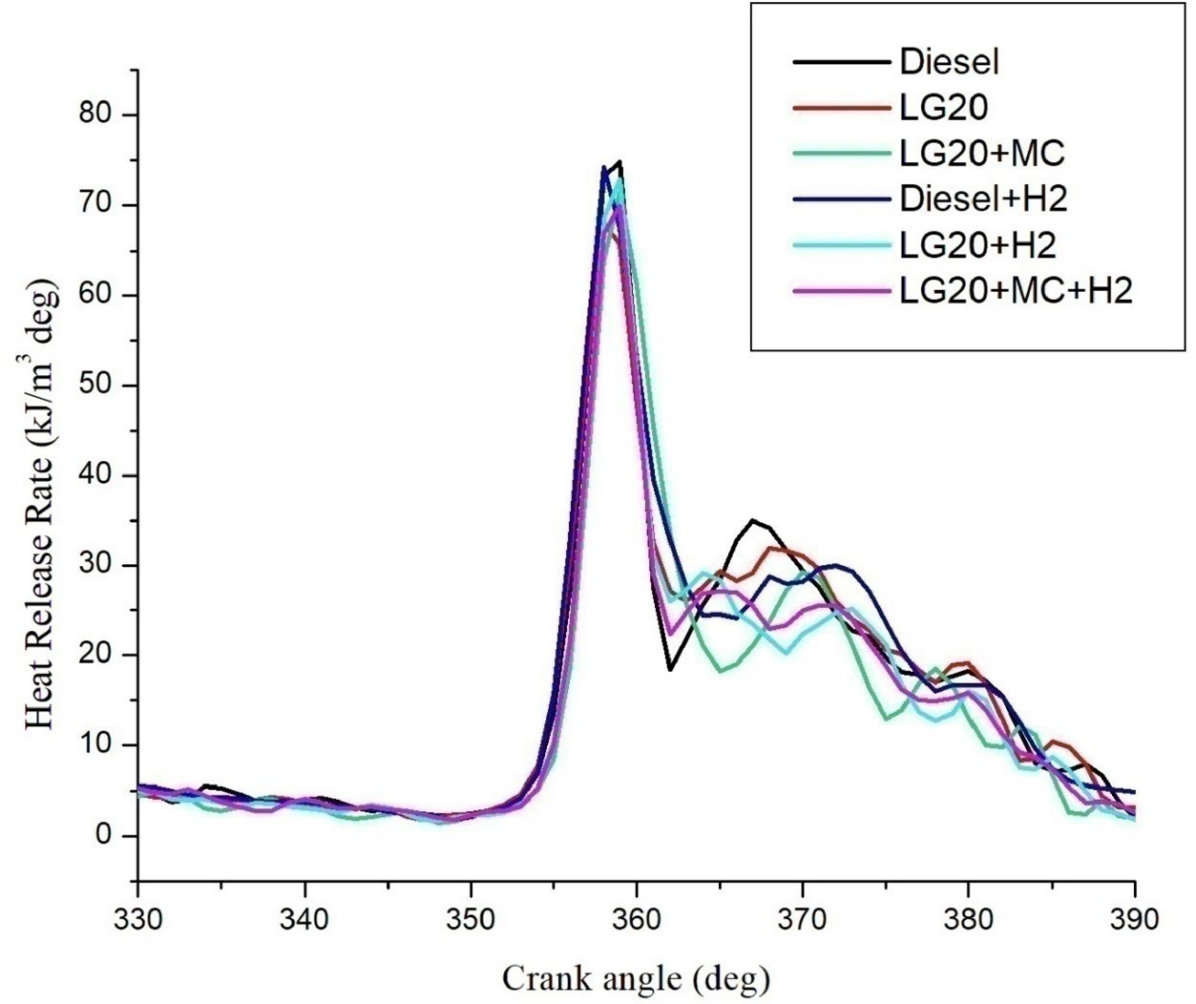
Distinctions of heat release rate and crank angle.

Figure 12 exhibits the differences in heat release rate for all fuels. Peak HRR was noted for LG20 + H_2_ fuel than for all samples of fuel. The combustion starts earlier for the LG20 fuel is the main reason for reducing the HRR for the LG20 fuel than the diesel fuel^36^. Improved combustion characteristics with MWCNTs nature, such as increased cetane number and larger surface-volume ratio, lead to an improvement in the HRR of the LG20 + MWCNT fuel compared to improve significantly than the LG20 fuel^24,26,28^. Hydrogen enrichment in LG20 fuel improves the flame front propagation and effectively burns the fuel sample, and also improves the HRR than the LH20 fuel^31,32^. The LG20 + MWCNT + H_2_ fuel enhances the HRR slightly due to the combined positive effects of MWCNTs and hydrogen gas^25,27–29^.

#### Exhaust gas temperature (EGT)

**Fig. 13:**
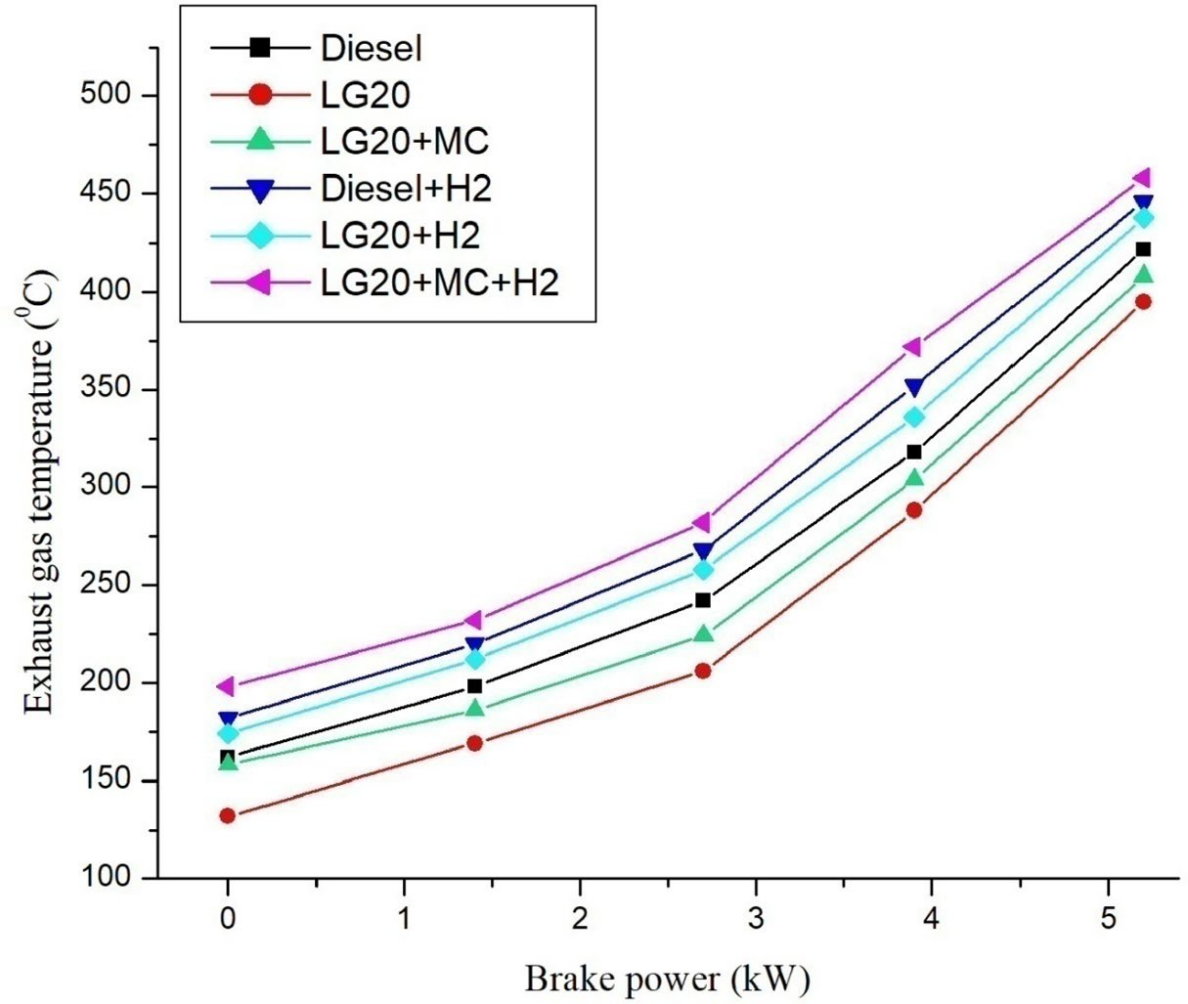
Distinctions of Exhaust gas Temperature and brake power.

Figure 13. depicts the variations of EGT with brake power. The maximum EGT was reported for the LG20 + MWCNT + H2 fuel at all testing conditions compared to all test fuels. The shortened ID period and reduced adiabatic flame temperature of biodiesel cause LG20 fuel to produce a lesser exhaust gas temperature (by 6.83%) than diesel fuel, but it increased by 3.29% with the dispersion of MWCNTs due to enhanced combustion efficiency. Introducing hydrogen into diesel and LG20 fuel, EGT was enhanced by 5.68% and 10.88%, respectively, compared to diesel and LG20 fuel samples. The combined impact of nano additive LG20 fuel with hydrogen mixing increases the EGT (by 12.25 and 4.56%) compared to LG20 + MWCNT and LH20 + H2 fuel samples^25,27–29^.

### Emission chatetaristics

#### Carbon monoxide (CO)

**Fig. 14:**
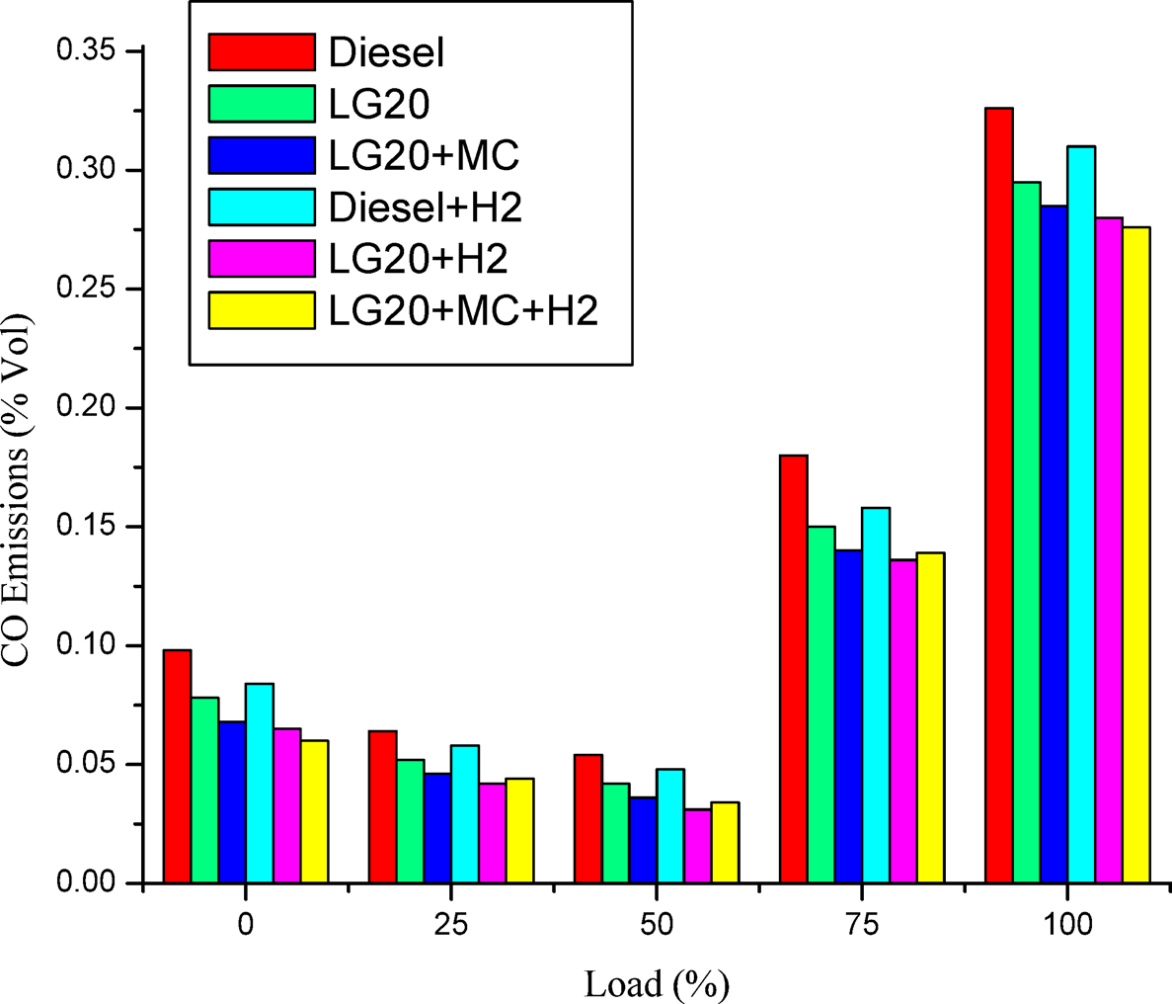
Distinctions of CO emissions and load.

Figure 14 depicts the variances in emissions of carbon dioxide with load for all tested fuels. The CO emission of LG20 fuel were diminished by 10.6% due to extra oxygen content; a large number of carbon molecules in the fuel were converted into carbon dioxide molecules compared to diesel^33,34^. Further reduction of CO emissions (by 3.5%) was noted with the dispersion of MWCNTs in LG20 fuel^24,26,28^. It is also noted that the CO emissions for the diesel + H2, LG20 + H2 fuels were reduced by 5.16% and 5.35% compared to diesel and LG20 fuels, respectively, because the intake air contains more quantity of hydrogen and oxygen, causing the formation of OH molecules, thereby reducing the CO emissions. The combined impact of MWCNTs and hydrogen addition into the fuel and intake air leads to lowered CO emissions by 6.88, 3.26%, and 1.5% compared to LG20, LG20 + MWCNT, and LG20 + MWCNT + H_2_ fuel samples, respectively^25,27–29^.

#### Carbon dioxide (CO_2_) emissions

**Fig. 15:**
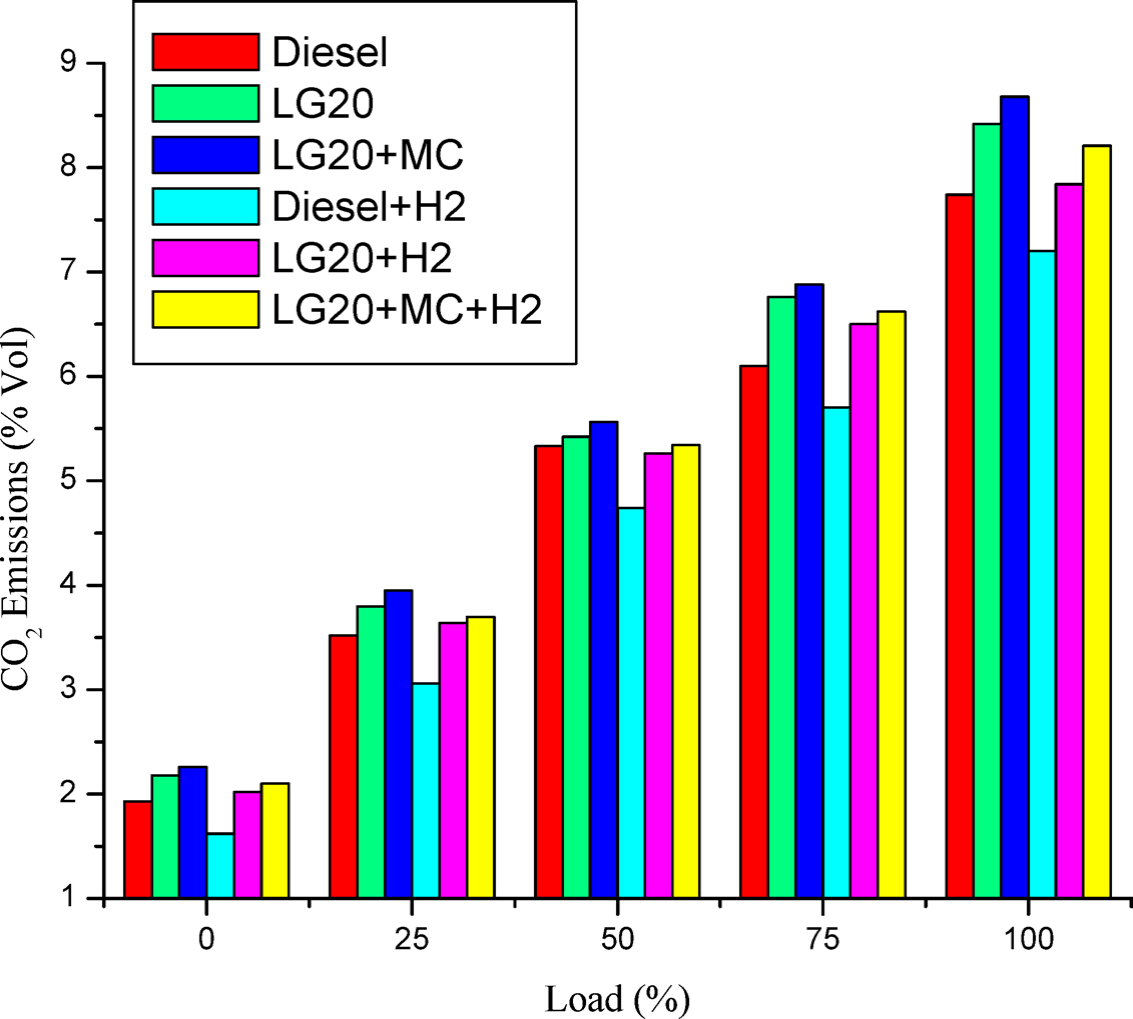
Distinctions of CO_2_ emissions and load.

Figure 15 describes the change in carbon dioxide (CO2) emissions with load for all fuels experimented. CO2 emissions have been seen to increase proportionally with load for all fuels experimented^35^. The CO_2_ emissions for LG20 and LG20 + MWCNT fuels were enhanced by 8.7% and 12.2% equated to diesel due to superior density and SOI (start of injection), but it can be compensated by providing H_2_ gas into an intake manifold by 1.29% with an increment of 7.39% compared to LG20 fuel. The engine operated with hydrogen inducted diesel fuel will exhibit the lowest CO_2_ emissions by 7.5%, 8.88%, and 16.9% compared to the diesel, LG20, and LG20 + H2 fuels, respectively. The LG20 + MWCNT + H_2_ fuel shows 2.55% and 5.72% of decrement in CO_2_ emissions compared to LG20 and LG20 + MWCNT fuels, respectively, because hydrogen addition promotes cleaner combustion^25,27–30^.

#### Hydrocarbon (HC) emissins

**Fig. 16:**
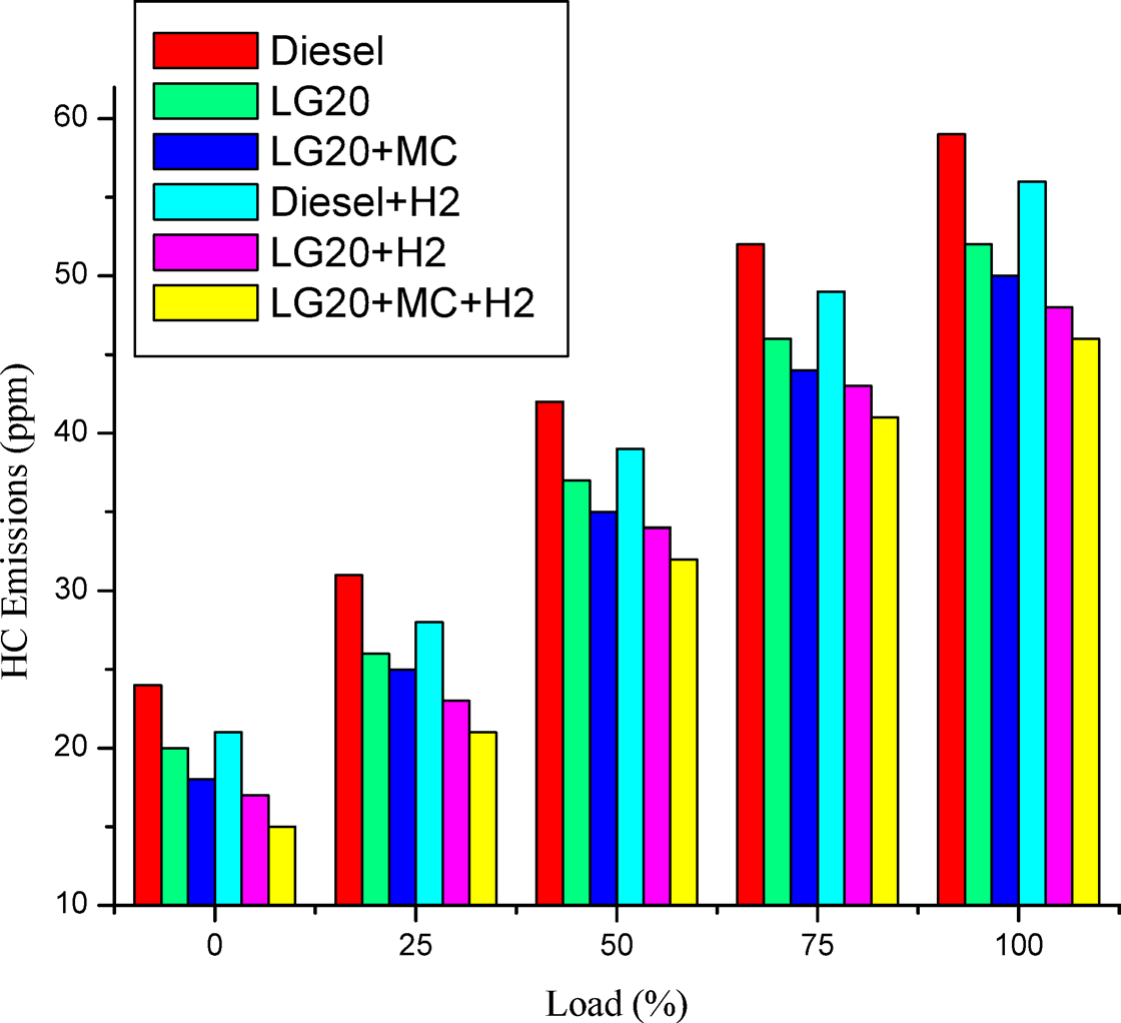
Distinctions of HC emissions and load.

Figure 16 displays the fluctuations of the release of hydrocarbons with a change in load for all test fuels. Because of biodiesel’s high oxygen content, the LG20 fuel emits 13.5% less HC than diesel^36^. Supplying hydrogen gas into an intake manifold while operating an engine with diesel and LG20 fuel, HC emissions were somewhat reduced by 5.35 and 8.33% in comparison to diesel and LG20 fuel, owing to the low carbon concentration in the fuel-air mixture. A slight reduction of HC emissions (by 4%) was noted with the LG20 + MWCNT fuel than the LG20 fuel due to improved combustion^37^. The lowest HC emissions were noted for LG20 + MWCNT + H2 fuel among all the fuels due to the collective effect of the addition of H_2_ and MWCNTs^25,27–29^.

#### NOx emissions

**Fig. 17:**
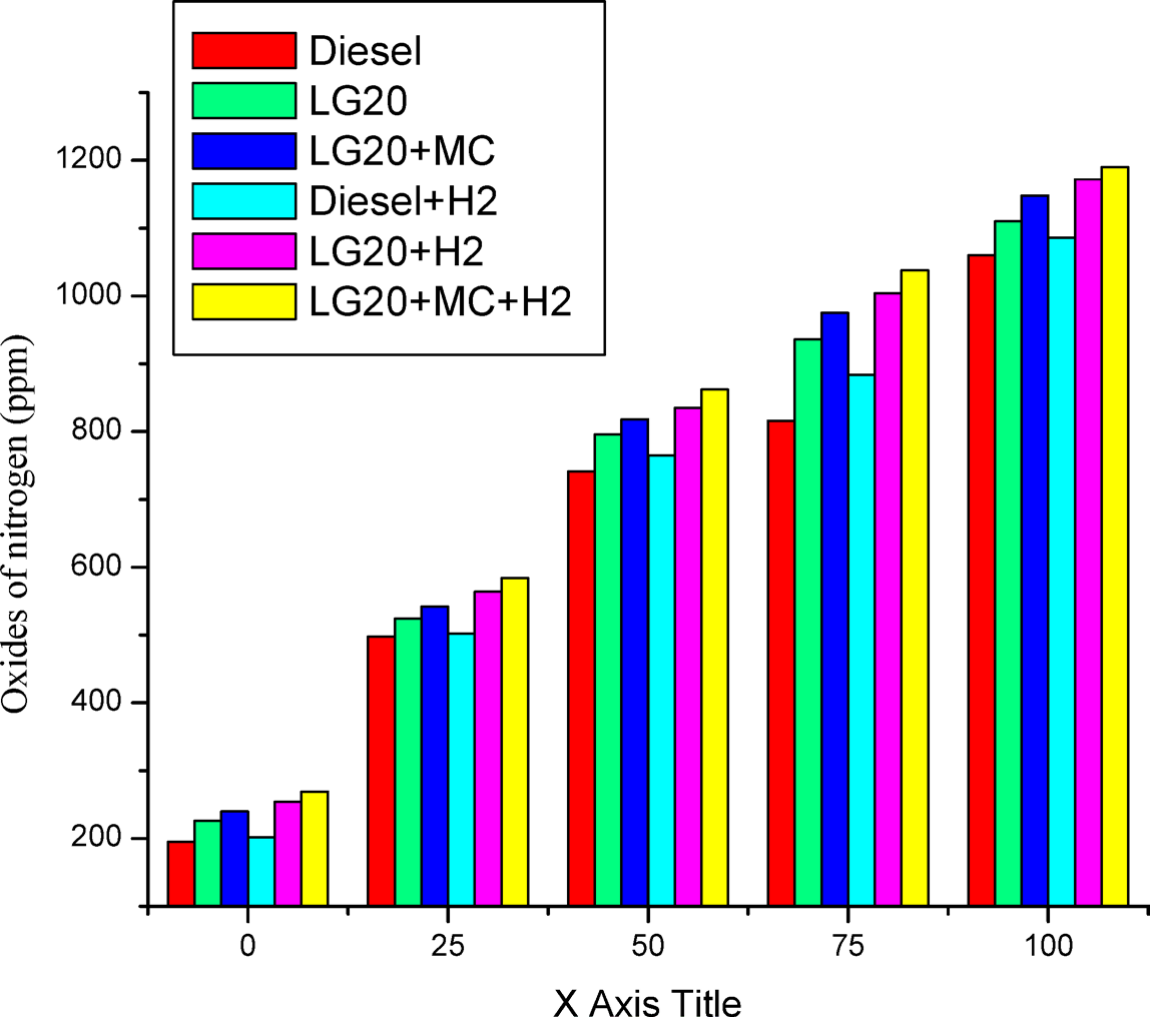
Distinctions of NO_x_ emissions and load.

Figure 17 depicts the fluctuations in Nox emissions according to the load with test fuels. The diesel’s emission levels of Nox were reduced by 4.7% relative to the LG20 fuel due to increased oxygen abundance and combustion temperature. LG20 + MWCNT and LG20 + H_2_ fuels increased Nox emissions by 3.42% and 5.5% when compared to the LG20 fuel, respectively, since MWCNTs promote fuel atomisation and H2 induction increases diffusion flame temperature during the combustion process. Hydrogen induction in diesel fuel improves the Nox emissions by 2.45% than diesel fuel because hydrogen has a higher calorific value, causing a rise in cylinder temperature. The Nox emissions for LG20, LG20 + MWCNT, and LG20 + H_2_ fuel were enhanced by 7.20%, 3.65%, and 1.53% compared to the LG20 + MWCNT + H_2_ fuel due to the adverse effects of MWCNTs and hydrogen induction to the LG20 fuel^25,27–29^.

#### Smoke opacity

**Fig. 18:**
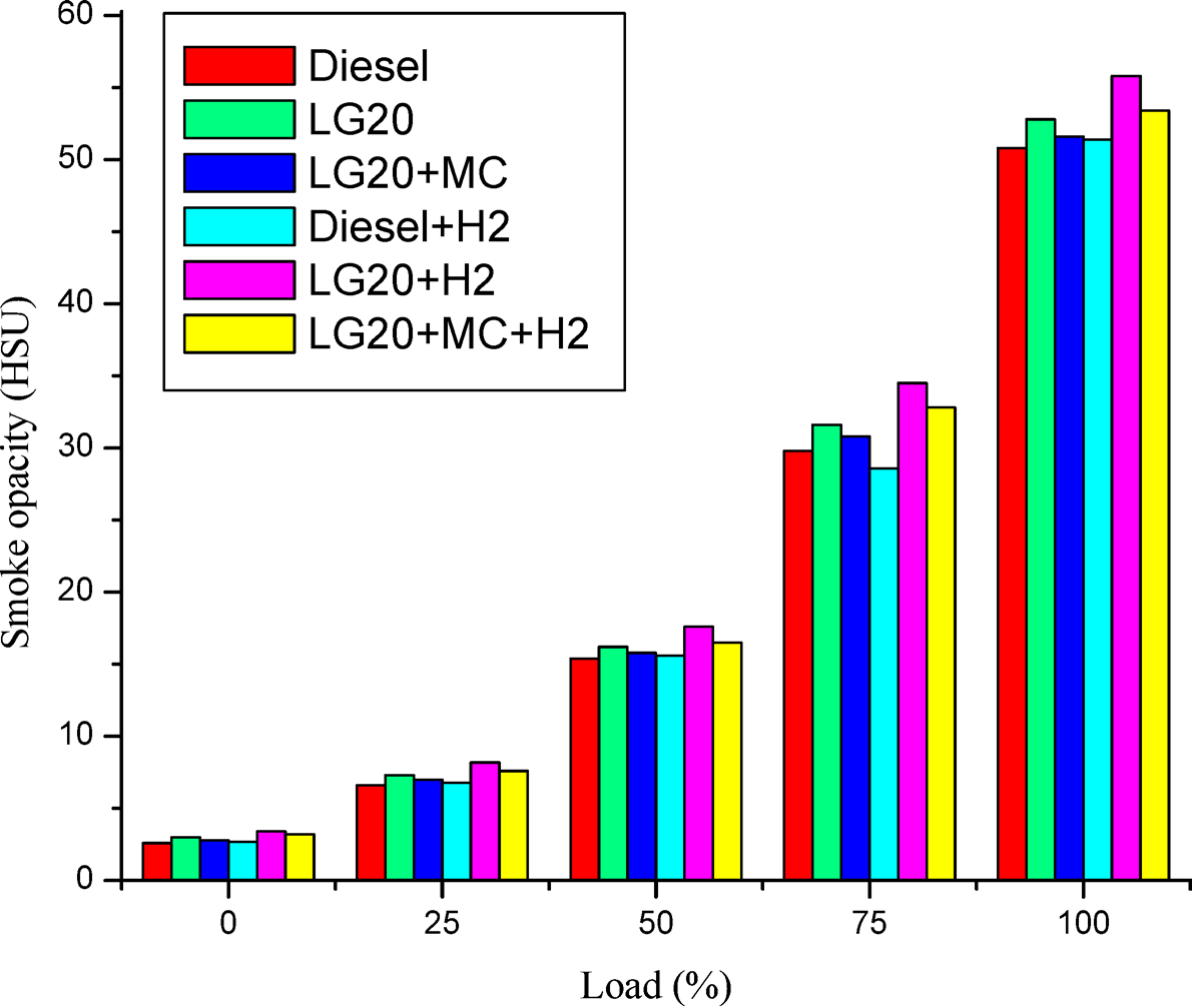
Distinctions of smoke emissions and load.

Figure 18 shows smoke opacity changes as a function of load using test fuels. Diesel and petrol produced the lowest smoke emissions of any fuel evaluated across all load conditions. The smoke opacity of LG20 fuel improved by 3.93% due to biodiesel’s increased density and reduced heating value compared to diesel^30,31^. This increase in smoke emissions was offset by a 2.32% reduction for the LG20 + MWCNT fuel at full load. The addition of hydrogen through an input manifold enhances the smoke opacity for diesel and LG20 fuels by 1.18 and 5.58%, respectively, over diesel and LG20 fuel. The smoke opacity for LG20 + MWCNT + H_2_ fuel was diminished by 4.49% than LG20 + H2 fuel, but increased by 1.13% and 3.48% compared to LG20 and LG20 + MWCNT fuels, respectively^24,26,28^.

## Conclusions

Applications for hydrogen in the home, workplace, and space include Using fuel cells to produce electricity. Individual Use: home energy, it is imperative that the scientific community move swiftly to discover solutions that will enable hydrogen energy to compete commercially with fossil fuels. The recent economic collapse has caused oil prices to decline, but if the trend continues, they will rise again, making it difficult for the average person to afford such high prices. Consequently, hydrogen emerges as a potential substitute for petroleum. Although its current cost is approximately four times higher than that of conventional fuels, hydrogen offers nearly three times greater energy efficiency; moreover, the transition toward hydrogen as a primary energy source is approaching, driven by rising crude oil prices and the decreasing cost of hydrogen production. All that needs to be done is to construct facilities for vehicles to be filled with hydrogen and implement safety measures for both stationary and mobile uses.

As a supplemental fuel replacement for diesel, the current study concentrated on non-edible 20% biodiesel derived from lemongrass (Cymbopogon Citratus) combined with diesel and enhanced with MWCNTs at 40 ppm in a CI engine without engine alteration. To improve the performance of this nano fuel even more, we premixed within the cylinder by adding hydrogen (5.0 LPM flow rate) to the entry section throughout the suction phase. 20% biodiesel yields lower performance due to a slight increase in Nox output. To address this issue, lemongrass biodiesel with the addition of MWCNTs was mixed with hydrogen gas, and the following findings were made:

- Adding hydrogen in the rate of 5 LPM to lemongrass biodiesel nano fuel increased brake thermal efficiency by 12.6%. This is because hydrogen gas burns faster, which speeds up the process of burning.
- It was further estimated that adding hydrogen (5 lpm) to 20% lemongrass biodiesel fuel, together with 40ppm MWCNT, will reduce the BSFC by 15.33% when compared to LG20. The LG20 + MWCNT + H2 fuel reduced blend operation by 2.32% as compared to diesel. This happens because fuels enhanced with hydrogen (H2) have a higher flame speed, which increases the combustion rate of the fuel mixtures.
- Introducing hydrogen into diesel and LG20 fuel, EGT was enhanced by 5.68 and 10.88%, respectively, compared to diesel and LG20 fuel samples. The collective impact of nano additives in LG20 fuel with hydrogen mixing increased EGT (by 12.25 and 4.56%) when compared to the LG20 + MWCNT and LH20 + H2 fuel samples.
- The combined effect of MWCNT and H_2_(LG20 + MWCNT + H_2_ fuel has 67.2 bar) exhibits a slight improvement in the peak pressure than LG20 + MWCNT (65.8 bar) and LG20 + H_2_(66.4 bar) fuel samples due to increased combustion rate and proper mixing of hydrogen gas in the atomized form of LG20 fuel. Hydrogen enrichment in LG20 fuel improves the flame front propagation.
- Hydrogen enrichment in LG20 fuel improves the flame front propagation and effectively burns the fuel sample, and also improves the HRR than the LH20 fuel. The LG20 + MWCNT + H_2_ fuel enhances the HRR marginally due to the combined beneficial impacts of MWCNTs and hydrogen gas.
- The dispersion of MWCNTs and induction of H2 in the LG20 fuel caused a small decrease due to quick combustion. The combined action of MWCNTs and hydrogen induction on LG20 fuel significantly minimizes the ignition delay over LG20 fuel.
- The combined action of MWCNTs & hydrogen inclusion into the fuel and intake air leads to lowered CO emissions by 6.88%, 3.26%, and 1.5% compared to LG20, LG20 + MWCNT, and LG20 + MWCNT + H2 fuel samples, respectively.
- The engine operated with hydrogen inducted diesel fuel will exhibit the lowest CO_2_ emissions by 7.5%, 8.88%, and 16.9% compared to the diesel, LG20, and LG20 + H2 fuels, respectively. The LG20 + MWCNT + H2 fuel shows a 2.55% and 5.72% of decrement in CO_2_ emissions compared to LG20 and LG20 + MWCNT fuels, respectively, because hydrogen addition promotes cleaner combustion.
- A slight decrease in HC emissions (by 4%) was observed with the LG20 + MWCNT fuel compared to the LG20 fuel, thanks to better combustion. Among all the fuels tested, the LG20 + MWCNT + H2 fuel showed the lowest HC emissions, due to the combined benefits of adding both hydrogen and MWCNTs.
- NOx emissions for LG20, LG20 + MWCNT, and LG20 + H2 fuel were enhanced by 7.20%, 3.65%, and 1.53% compared to the LG20 + MWCNT + H2 fuel, respectively, due to the adverse effects of MWCNTs and hydrogen induction on the LG20 fuel.
- Adding hydrogen into the intake manifold increases smoke opacity in diesel and LG20 fuels by 1.18% and 5.58%, respectively. The smoke opacity for LG20 + MWCNT + H2 fuel was diminished by 4.49% than LG20 + H2 fuel, but increased by 1.13% and 3.48% compared to LG20 and LG20 + MWCNT fuels, respectively.

The findings suggest that hydrogen port injection methods and lemon grass biodiesel blends with MWCNT additives can be efficiently employed in dual-fuel diesel engines to produce electricity in stationary engines. Future research can focus on LG20 + MWCNT + H2 paired using SCR and EGT approaches to regulate NOx in exhaust while maintaining diesel engine performance.

## Data Availability

The datasets used and/or analyzed during the current study are available from the corresponding author on reasonable request.

## Abbreviations

TDC: Top dead Centre
TBC: Thermal barrier coating
RSM: Response surface methodology
ppm: Parts per million
NOx: Oxides of nitrogen
H_2_: Hydrogen
HC: Hydrocarbon
GCMS: Gas chromatography–mass spectrometry
FTIR: Fourier transform infrared
EGT: Exhaust gas temperature
DFM: Dual fuel mode
DI: Direct injection
CA: Crank Angle
CI: Compression Ignition
CO: Carbon monoxide
BSFC: Brake-specific fuel consumption
BTE: Brake thermal efficiency
bTDC: Before Top Dead Center
ASTM: American Society for Testing and Materials
JB20: 20% Jojoba biodiesel + 80% diesel
